# Exploring the impact of stress on the electronic structure and optical properties of graphdiyne nanoribbons for advanced optoelectronic applications

**DOI:** 10.1038/s41598-024-56380-z

**Published:** 2024-03-13

**Authors:** Qiaohan Liu, Naixing Feng, Yi Zou, Chuanqiang Fan, Jingang Wang

**Affiliations:** 1 College of Science, Liaoning Petrochemical University, Fushun, 113001 China; 2 Key Laboratory of Intelligent Computing and Signal Processing, and School of Electronic and Information Engineering, Anhui University, Hefei, 230601 China

**Keywords:** Nanoscale materials, Electronic properties and materials, Two-dimensional materials

## Abstract

Graphdiyne (GDY), a two-dimensional carbon material with sp- and sp^2^-hybridization, is recognized for its unique electronic properties and well-dispersed porosity. Its versatility has led to its use in a variety of applications. The precise control of this material's properties is paramount for its effective utilization in nano-optical devices. One effective method of regulation, which circumvents the need for additional disturbances, involves the application of external stress. This technique provides a direct means of eliciting changes in the electronic characteristics of the material. For instance, when subjected to uniaxial stress, electron transfer occurs at the triple bond. This results in an armchair-edged graphdiyne nanoribbon (A(3)-GDYNR) with a planar width of 2.07 nm, which exhibits a subtle plasmon effect at 500 nm. Conversely, a zigzag-edged graphdiyne nanoribbon (Z(3)-GDYNR) with a planar width of 2.86 nm demonstrates a pronounced plasmon effect within the 250–1200 nm range. This finding suggests that the zigzag nanoribbon surpasses the armchair nanoribbon in terms of its plasmon effect. First principles calculations and ab initio molecular dynamics further confirmed that under applied stress Z(3)-GDYNR exhibits less deformation than A(3)-GDYNR, indicating superior stability. This work provides the necessary theoretical basis for understanding graphene nanoribbons (GDYNRs).

## Introduction

Carbon is one of the most abundant elements in nature and is capable of forming stable and highly flexible bonds in low-dimensional materials. The different structures and properties of carbon materials are due to these different carbon–carbon bonds, such as sp^3^, sp^2^, and sp hybrids. Carbon atoms form various allotrope through these three hybrid states, including sp^3^ hybrid diamond^1,2^, sp^3^ and sp^2^ hybrid carbon nanotubes^3–7^, fullerenes^8–11^, and graphene^12–15^. GDY^16–23^ is characterized by triple bonds formed by sp hybridization, and has linear structure, no cis–trans isomeric, and high conjugation^24,25^. Among them, the performance of surface plasmon about two-dimensional GDY materials is comparable to that of metal surfaces. These properties give it excellent electrical, optical and photoelectric properties, making it a key material for the next generation of electronic optoelectronic devices. Due to its superior performance, GDY-family nembers have attracted the attention of many scientists^26,27^.

In theory, quasi-one-dimensional GDYNRs are obtained by tailoring two-dimensional GDY^28^. Among them, GDYNRs is a one-dimensional material with uniform edges and nanoscale width. Depending on the boundary structure, GDYNRs can be divided into armchair edge graphdiyne nanoribbons (A(n)-GDYNRs) and zigzag edge graphdiyne nanoribbons (Z(n)-GDYNRs). The characteristics of GDYNRs are greatly affected by changes in edge structure and width, so the characteristics of GDYNRs can be adjusted by various shear attempts on the GDY plane. Until 2020 that Li's team made an important breakthrough in the preparation of GDYNR. A "two-step" strategy for synthesizing GDYNRs has been successfully reported^29^. The first chemically synthesized GDYNRs was prepared by this method. They consist of diamond-shaped elements, with benzene as the vertex and "$- C \equiv C - C \equiv C -$" as the edge, and are determined to be about 4 nm in width and several hundred nm in length^29^. Subsequently, in order to further explore the stability of materials, many researchers used AIMD simulation to conduct stability analysis of materials^30,31^. Li et al. further explored the stability of GDYNRs through AIMD simulations. AIMD simulation results show that the total energy fluctuation of GDYNRs is very small, and the average total energy fluctuation is less than 0.439%^32^.

At the same time, a large number of theoretical studies have been carried out to elucidate the relationship between their structure and properties^33–37^. These studies include tailoring two-dimensional nanosheets into one-dimensional nanoribbons of different widths and configurations, stacking multiple layers of GDYNRs, and doping atoms to adjust the band structure and thus change the photoelectric properties of the materials^34,38^. In addition, the band structure of one-dimensional nanoribbons and two-dimensional nanosheet can also be adjusted by stress^39–42^. The research found that, the stress induced transformation of two-dimensional materials from indirect to direct band gap greatly improves the luminous efficiency and makes them more suitable for optoelectronic devices^43–52^. A large number of studies have shown that the adjustable band gap of GDY increases monotonically with the uniform increase of stress^53–56^. Therefore, stress adjustable novel nano-electronic and optoelectronic devices have great application potential. However, the band gap response of GDYNRs to uniaxial tensile and compressive stress has rarely reported. To gain insight into this, in this work, the photoelectric properties of A(3)-GDYNR and Z(3)-GDYNR under uniaxial tensile and compressive stress are studied by first principles method.

## Methods

**Figure 1:**
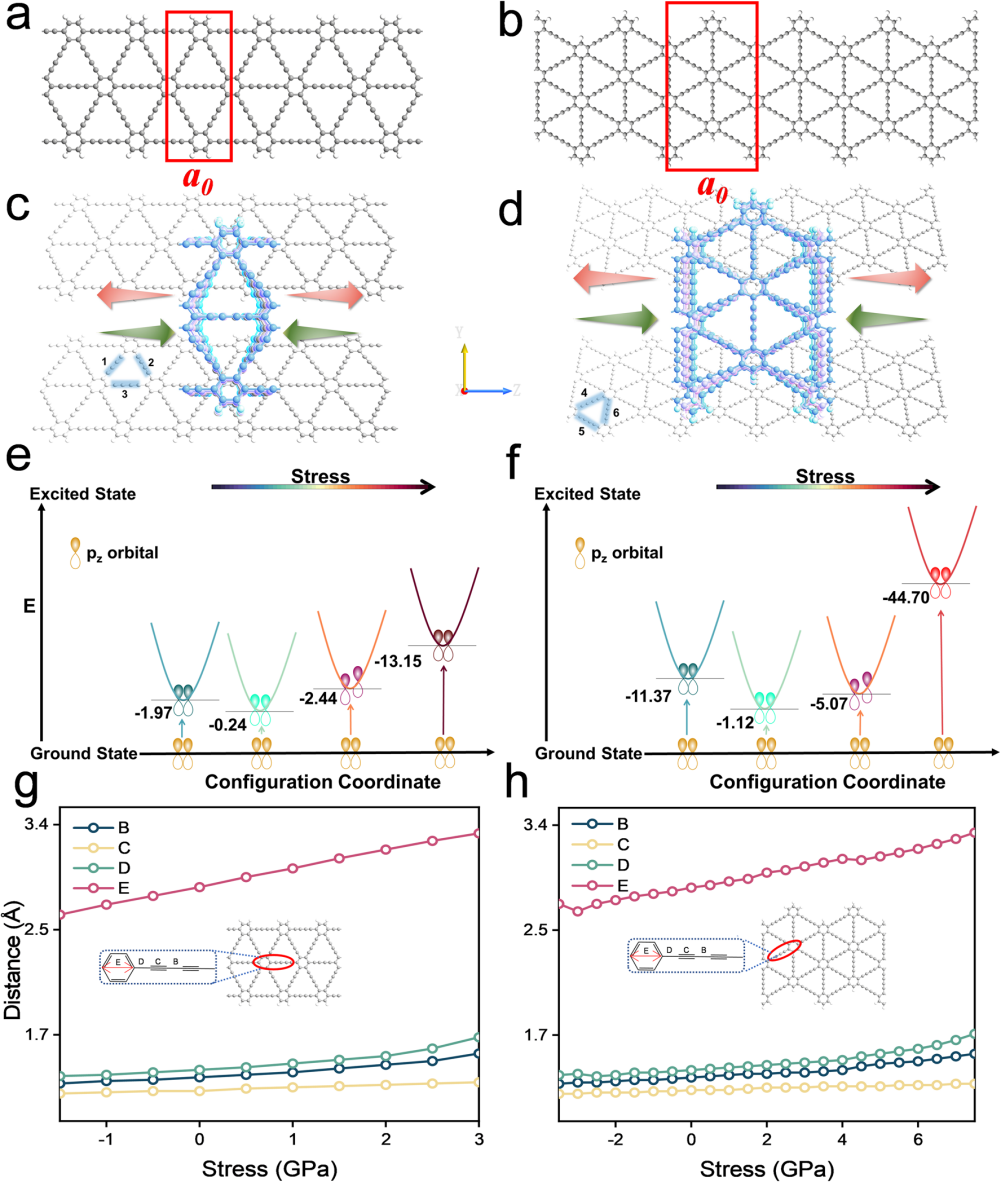
(**a**) Models of one-dimensional nanoribbons with armchair edges (A(3)-GDYNR); (**b**) zigzag edges (Z(3)-GDYNR); (**c**) structure diagram of A(3)-GDYNR under stress of − 1.5 GPa, − 1 GPa, 0 GPa, 1 GPa, 1.5 GPa, and 3 GPa; (**d**) structure diagram of Z(3)-GDYNR under stress of − 3.5 GPa, − 2 GPa, 0 GPa, 2 GPa, 3.5 GPa and 7.5 GPa; (**e**, **f**) p_z_ orbit diagram of energy and stress required for A(3)-GDYNR and Z(3)-GDYNR from ground state to first excited state; (**g, h**) the change of bond length of A(3)-GDYNR and Z(3)-GDYNR structure under different stress.

In this work, the QuantumATK-2019 package (fermitech, Beijing, China)^57^ was used to calculate two quasi-one-dimensional GDYNRs of different widths and configurations (A(3)-GDYNR and Z(3)-GDYNR). For the A(n)-GDYNRs structure, it extends in the same direction as the two chains of carbon atoms, at a 30° from the other two chains of carbon atoms. The extension direction of the nanoribbons in the Z(n)-GDYNRs structure is at a 30° or perpendicular to the arrangement direction of the carbon atom chains (Fig. 1). Where A(3)-GDYNR represents nanoribbons with an edge width of 3 hexagonal carbons (with widths n = 3), and Z(3)-GDYNR represents nanoribbons with an edge width of 3 hexagonal carbons (with widths n = 3).

As shown in Fig. 1a, b, to avoid dangling keys at the edges. The hanging bonds of carbon atoms on the edge of the tailoring nanoribbon are saturated with hydrogen, and repeat unit cells are in the red wire frame. The number of atoms in unit cells of A(3)-GDYNR and Z(3)-GDYNR are 50 and 100, respectively. The cell parameters of the structure of A(3)-GDYNR (width of repeating cells along the nanoribbon extension direction Z) is ${\text{a}}_{0} = 0.946nm$. The ZGDYNRs cell parameter is ${\text{a}}_{0} = 1.639\;{\text{nm}}$. The widths of A(3)-GDYNR and Z(3)-GDYNR restricted directions are 2.07 nm and 2.86 nm, respectively. The basis set was linear combination of atomic orbitals (LCAO), pseudo potentials were determined by Pseudo Dojo^58^ and electronic exchange–correlation potential was treated by the generalized gradient-Perdew Burke Ernzerh of functional (GGA-PBE), combined with the density functional theory with Grimme’s D3 correction and without damp (DFT-D3) dispersion correction and DFT-D3 can better perform van der Waals correction^59^. In the calculations, the DFT-LCAO module is employed with 50 Hartree cut-off energy, which combines the pseudopotential with the linear combination of atomic orbitals. The convergence criterion for the total energy is set at 10^−5^ eV and the K-mesh was 1 × 1 × 3. A vacuum layer of 15 Å is applied in the direction perpendicular to the ribbon plane and between the neighboring ribbons.

In order to study the effects of uniaxial tensile and compressive stress on the stability and electrical properties of GDYNRs. AIMD simulations of A(3)-GDYNR and Z(3)-GDYNR under stress induction were performed. The QuantumATK-2019 package (fermitech, Beijing, China)^57^ was used to adopt PBE functional and DFT-D3 correction method^59^. Under constant volume conditions, the simulation with time step of 1 fs and a total of 1 ps was carried out at 300 K and 600 K respectively.

**Figure 2:**
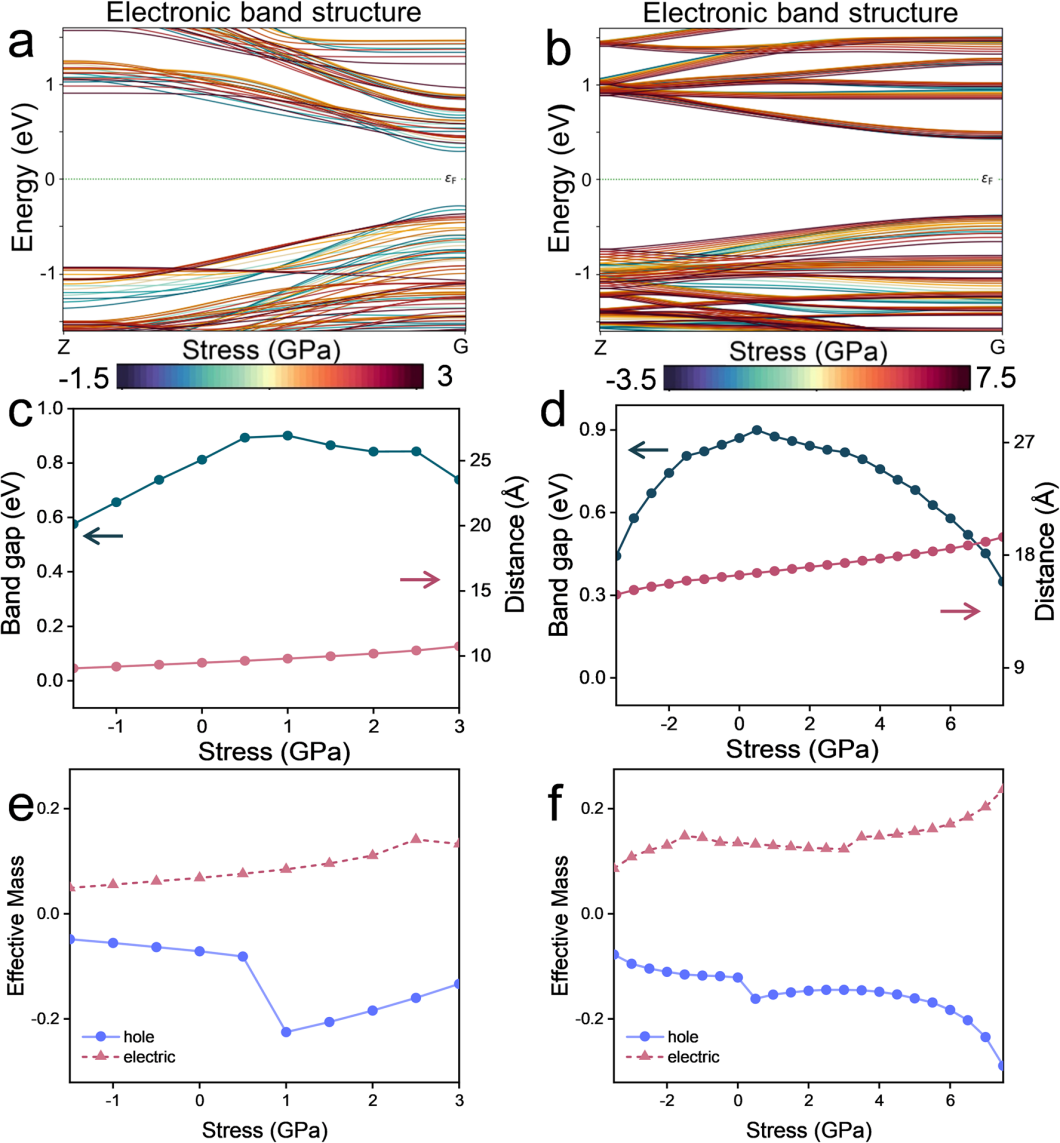
(**a**) Electron band structure of A(3)-GDYNR under different stress (− 1.5 to 3.GPa); (**b**) electron band structure of Z(3)-GDYNR under different stress (− 3.5 to 7.5 GPa); (**c, d**) A(3)-GDYNR and Z(3)-GDYNR band gap changes under different stress (green line), cell parameter a_0_ changes with different stress (red line); (**e, f**) plots of the effective mass of A(3)-GDYNR and Z(3)-GDYNR with different stress applied perpendicular to the plane.

The stress is applied along the Z direction (vertical the plane direction). The direction of the arrow indicates the direction of the applied stress (Fig. 1c, d). Where, the red arrow represents the uniaxial tensile stress applied to the nanoribbon, and the green arrow represents the uniaxial compressive stress applied to the nanoribbon. In order to more intuitively observe the expansion and contraction of the structure. In this paper, the Z direction is denoted as vertical the plane direction, and the Y direction is denoted as in-plane direction (Fig. 1). Figure 2c, d visualizes the variation of the unit cell parameter a_0_ of A(3)-GDYNR and Z(3)-GDYNR with different stress.

## Results and discussion

In this work, the optical properties of A(3)-GDYNR and Z(3)-GDYNR (Fig. 1a, b) structures under applied stress modulation are theoretically studied. Initially, the stability of these two structures is evaluated. The energy required from the ground state of A(3)-GDYNR and Z(3)-GDYNR to the first excited state under different stress are calculated. The energy values of two structures at 0 GPa are used as reference. Figure 1e, f respectively showed the p_z_ orbit diagrams of the energy and stress required by A(3)-GDYNR and Z(3)-GDYNR from the ground state to the first excited state. It can be obviously observed that the energy of both structures increases when compressive or tensile stress are applied perpendicular to the plane direction. In other words, the stability of the two structures decreases with the increase of the stress. Although the stability of the two structures changes in the same trend under the stress conditions, there are still differences. This depends on the structure of the material and the amount of applied stress. When uniaxial compressive stress (− 1.5 to 0 GPa) is applied to A(3)-GDYNR in the vertical plane direction, the maximum energy required is 1.97 eV. And uniaxial tensile stress (0–3 GPa) is applied, the maximum energy required is 13.15 eV. At the same time, when Z(3)-GDYNR is subjected to uniaxial compressive stress (− 3.5 to 0 GPa) perpendicular to the plane, the maximum energy required is 11.37 eV. However, when uniaxial tensile stress (0–7.5 GPa) is applied, the maximum energy required is 44.70 eV.

In order to further study the stability of A(3)-GDYNR and Z(3)-GDYNR. The AIMD simulation is carried out in this work. The results showed that the A(3)-GDYNR and Z(3)-GDYNR structures maintained well without obvious distortion or collapse under the influence of stress (Fig. S1a, Fig. S1d), and the difference of bond length before and after AIMD simulation was less than 0.06 Å. With the increase of tensile and compressive stress, the motion amplitude of A(3)-GDYNR and Z(3)-GDYNR increases (Fig. 1). With the increase of stress, The root-mean-square deviation (RMSD) curves of the A(3)-GDYNR and Z(3)-GDYNR tracks both showed an upward trend (Fig. S3a–S3b). It is worth noting that the RMSD curve keeps rising for A(3)-GDYNR with applied stress of 3 GPa, A(3)-GDYNR with applied stress of − 1.5 GPa, and Z(3)-GDYNR with applied stress of 7.5 GPa and − 3.5 GPa. However, other RMSD trajectories have peaks. The repeated rise and fall of RMSD indicates that it can carry out periodic vibration and can exist stably.

In addition, the AIMD simulations of A(3)-GDYNR and Z(3)-GDYNR at 300 K and 600 K are also studied. With the increase of temperature, RMSD curves of A(3)-GDYNR and Z(3)-GDYNR tracks both showed an upward trend (Fig. S3a–S3b). The RMSD curve shows that the higher the simulated temperature, the greater the geometric fluctuation caused by thermal motion. This phenomenon can be directly observed in Fig. S2. In order to further confirm the stability of the structure, the phonon spectra of A(3)-GDYNR and Z(3)-GDYNR are also calculated. There are no virtual frequencies in the phonon spectrum (Fig. S4).

Subsequently, the effect of uniaxial stress on bond length in A(3)-GDYNR and Z(3)-GDYNR structures was investigated (Fig. 1g, h). It can be observed that the acetylene bonds of the two structures (C in Fig. 1g, h) are least affected by external stress. In the A(3)-GDYNR structure, the bond lengths of E (2.64–3.29 Å), B (1.29–1.53 Å) and D (1.35–1.66 Å) increase significantly with the uniaxial stress (− 1.5 to 3 GPa). At the same time, the interaction forces between the atoms connected by these stretched bonds also change. As a result, the ground state energy at the bottom of the conduction band (CB) decreases, while the ground state energy at the top of the valence band (VB) increases (Fig. 2a). The specific performance is shown in Fig. 2c. With the increase of stress, the band gap of A(3)-GDYNR decreases. It is worth noting that under the same external stress, Z(3)-GDYNR changes less significantly than A(3)-GDYNR (Fig. 1h). Changes in the bond lengths of E (2.67–3.30 Å), B (1.29–1.53 Å), and D (1.36–1.69 Å) in the Z(3)-GDYNR structure require greater stress (− 3.5 to 7.5 GPa) to be applied relative to A(3)-GDYNR. This is because the "$- C \equiv C - C \equiv C -$" in the ZGDYNR structure is not parallel to the direction in which the stress is applied. The applied stress mainly affects the bond Angle but not the bond length of the ZGDYNRs structure. This also causes the ground state energy of Z(3)-GDYNR CB bottom and VB top to change little compared with A(3)-GDYNR under the same external stress. Specifically, under the influence of the same external stress, the band gap of Z(3)-GDYNR decreases less significantly than that of A(3)-GDYNR with the increase of stress (Fig. 2d).

Figure 2a–d show the band structure of these two structures near Fermi level and the variation trend of band gap under corresponding applied stress. In Fig. 2a, b, A(3)-GDYNR and Z(3)-GDYNR present a direct band gap at the Gamma point. This work presents a way of modulating the band gap, that is, applying stress externally. The maximum energy of valence band (VBM) and minimum energy of conduction band (CBM) of the structure change with the change of applied stress. The structural band gap can be adjusted by the applied stress. The calculated bandgaps of A(3)-GDYNR and Z(3)-GDYNR without applied stress are 0.813 eV and 0.887 eV respectively. It is in good agreement with previous results^36^. The results show that the band gap of A(3)-GDYNR and Z(3)-GDYNR can be regulated by applied stress.

To obtain the quantitative scaling of band gap ($E_{{\text{g}}}$) with respect to the compressive and tensile stress. The functional relationship between band gap and applied stress is studied in this work. Figure S5 shows the functional relationship between the band gap of A(3)-GDYNR , Z(3)-GDYNR and applied compressive , tensile stress. We fit the corresponding data of A(3)-GDYNR and Z(3)-GDYNR into $E_{{\text{g}}} = E_{0} + a_{1} \delta + b_{1} \delta^{2}$ respectively. Where $E_{0}$ represents the A(3)-GDYNR and Z(3)-GDYNR bandgaps without external force. $a_{1}$ and $b_{1}$ of A(3)-GDYNR under compressive stress were 0.173 and 0.050, respectively. $a_{1}$ and $b_{1}$ of A(3)-GDYNR under tensile stress are 0.048 and − 0.029, respectively. $a_{1}$ and $b_{1}$ of Z(3)-GDYNR under compressive stress are 0.020 and 0.039, respectively. The $a_{1}$ and $b_{1}$ of Z(3)-GDYNR under tensile stress are 0.014 and − 0.011, respectively.

It is worth noting that coefficients $a_{1}$ and $b_{1}$ in equation $E_{{\text{g}}} = E_{0} + a_{1} \delta + b_{1} \delta^{2}$ reflect the sensitivity of A(3)-GDYNR and Z(3)-GDYNR to stress changes^37^. Under the regulation of compression (Fig. S5a, Fig. S5c) and tensile stress (Fig. S5b, Fig. S5d), the coefficients $a_{1}$ and $b_{1}$ of A(3)-GDYNR are always greater than Z(3)-GDYNR. This further indicates that the band gap change of A-GDYNRs is more affected by external forces than that of Z-GDYNRs.

The effective masses of the two structures with stress are also calculated (Fig. 2e, f). When no external stress is applied, it can be observed that the effective masses of electrons (0.086 m_0_) and holes (-0.087 m_0_) in A(3)-GDYNR are smaller than those of electrons (0.174 m_0_) and holes (-0.149 m_0_) in Z(3)-GDYNR. The conduction mode of A(3)-GDYNR was analyzed. It is found that the effective masses of electrons and holes of A(3)-GDYNR are equivalent when the applied stress is − 1.5 to 0.5 GPa. The electrons and holes conduct electricity at the same time (Fig. 2e). When the externally applied stress exceeds 0.5 GPa, the effective mass of the hole dominates. In this case, hole conduction is dominant. However, the conduction mode of Z(3)-GDYNR is different from that of A(3)-GDYNR. When the externally applied stress is − 3.5 to 0 GPa, the effective mass of the electron dominates. And when the external stress is 0–7.5 GPa, the effective mass of the hole dominates. The band gap of A(3)-GDYNR is almost linearly regulated when the external stress is between − 1.5 and 0.5 GPa. The band gap of Z(3)-GDYNR is less affected by stress regulation than that of A(3)-GDYNR.

**Figure 3:**
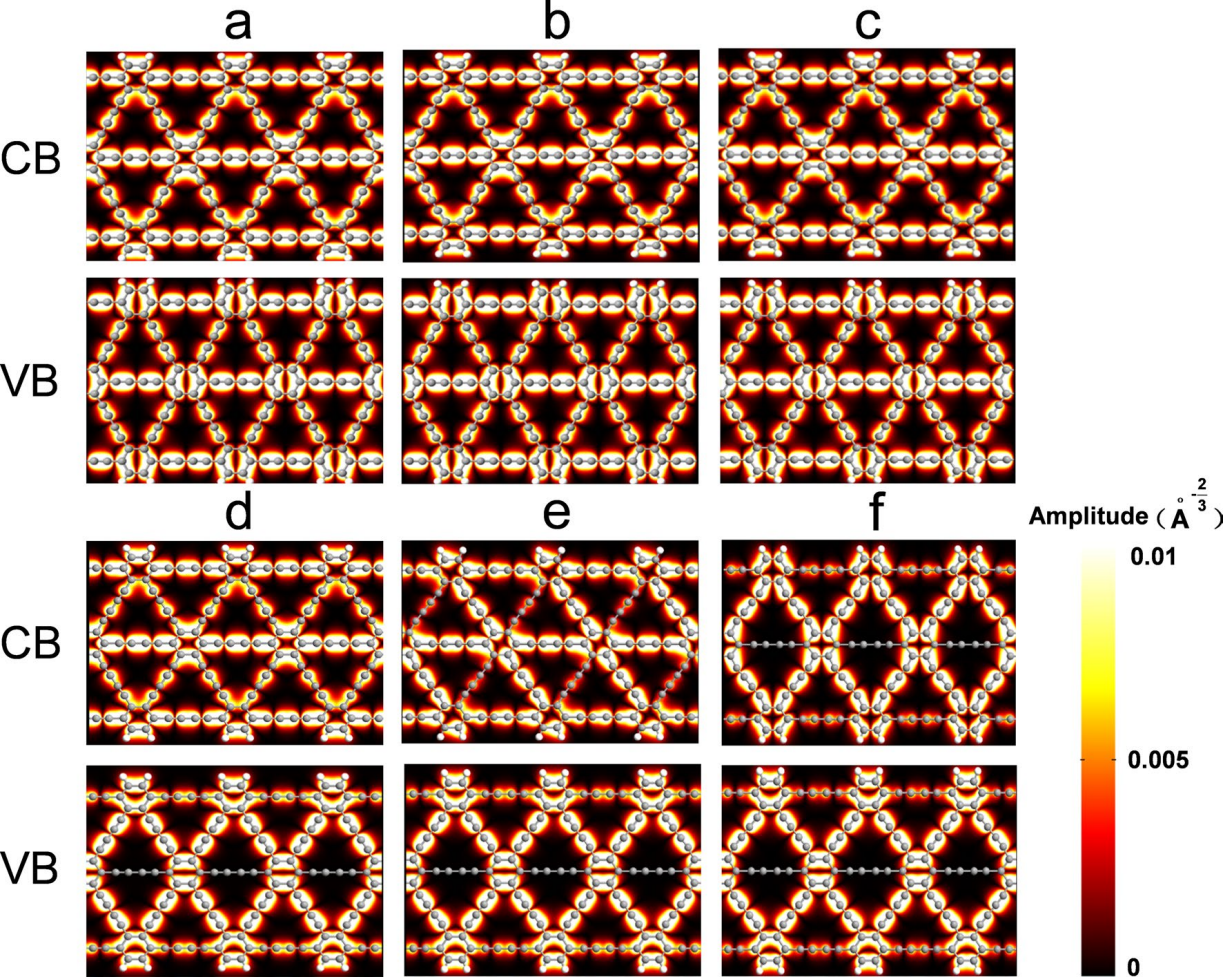
(**a**) A(3)-GDYNR show the bloch states of the CB (up), VB (down) at the gamma point. (**a**) − 1.5 GPa; (**b**) − 1 GPa; (**c**) 0 GPa; (**d**) 1 GPa; (**e**) 2.5 GPa; (**f**) 3 GPa.

**Figure 4:**
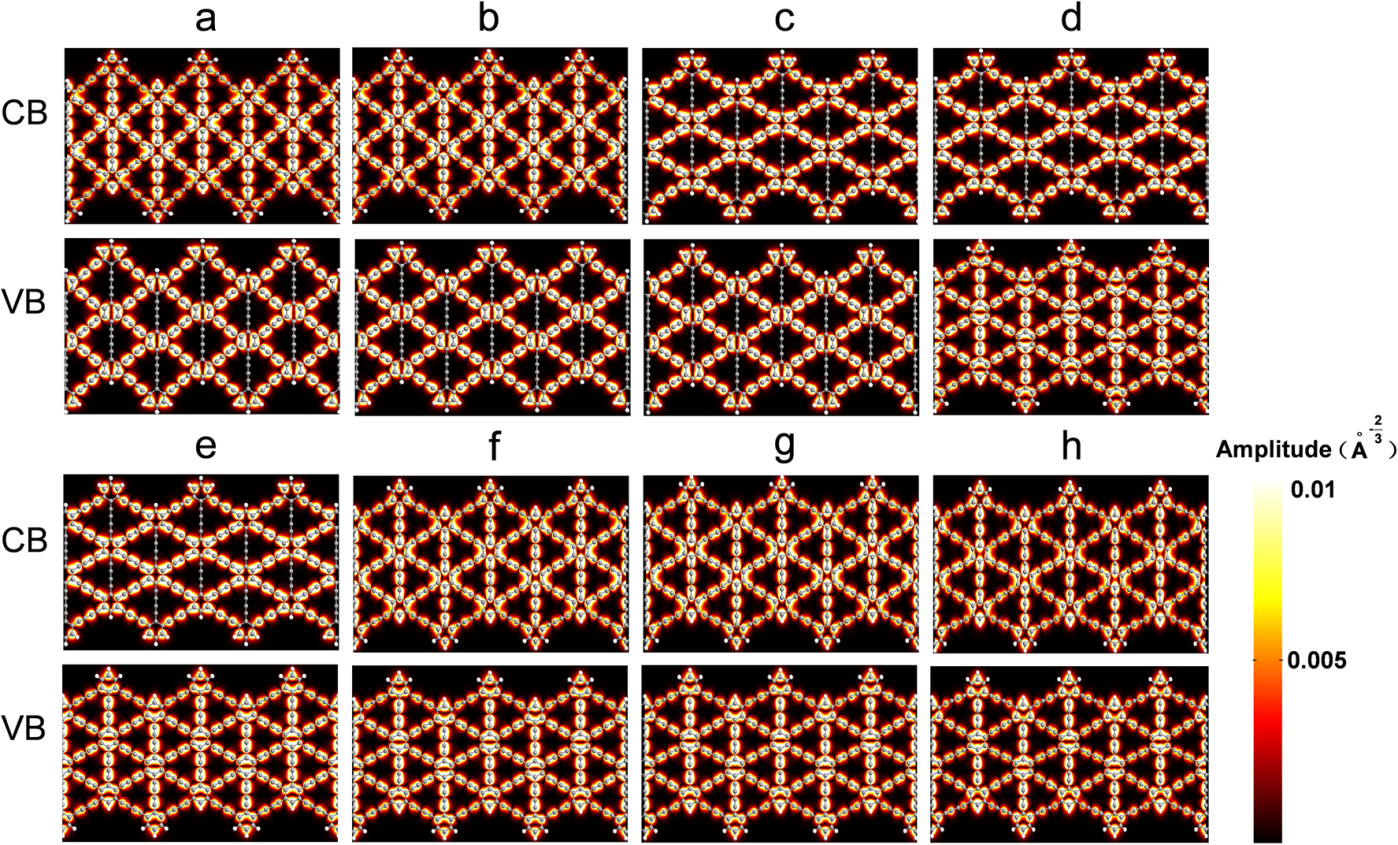
Z(3)-GDYNR show the bloch states of the CB (up), VB (down) at the gamma point. (**a**) − 3.5 GPa; (**b**) − 2 GPa; (**c**) 0 GPa; (**d**) 0.5 GPa; (**e**) 2 GPa; (**f**) 3.5 GPa; (**g**) 5GPa; (**h**) 7.5 GPa.

Subsequently, the influence of external uniaxial tensile stress on the electronic properties of GDYNRs is discussed. Figures 3 and 4 show Bloch wave functions of A(3)-GDYNR and Z(3)-GDYNR under CB and VB respectively. The Bloch wave functions of VB and CB are redistributed as the externally applied stress changes. With respect to CB, it can be observed that the orbits around the aromatic rings of the two structures are not completely delocalized. In the case of VB, electrons are mainly distributed around the benzene ring and "$- C \equiv C - C \equiv C -$"the number of 1, 2, 3. It can be observed that the carbon atoms in the benzene ring at VB have bonding characteristics with the carbon atoms "$- C \equiv C - C \equiv C -$"at the number of 1, 2, and 3. It can be observed that the delocalization of the electronic states at VB of A(3)-GDYNR at 1, 2, 3 "$- C \equiv C - C \equiv C -$" is much larger than that of Z(3)-GDYNR. This is because the nanosheets are cut in different directions into zigzag and armchair nanoribbons. Therefore, the difference of marginal morphology leads to the appearance of this phenomenon.

**Figure 5:**
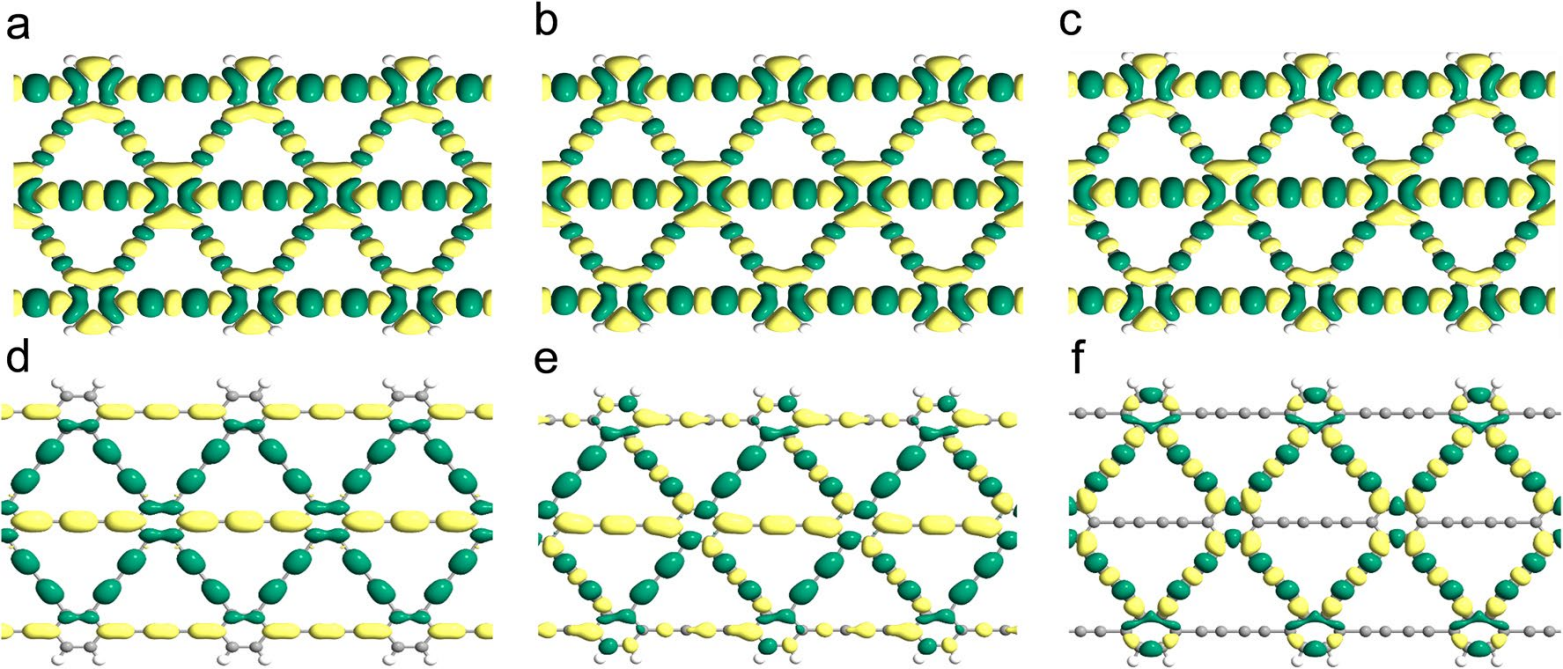
CDD of A(3)-GDYNR at the gamma point. (**a**) − 1.5 GPa; (**b**) − 1 GPa; (**c**) 0 GPa; (**d**) 1 GPa; (**e**) 2.5 GPa; (**f**) 3 GPa.

**Figure 6:**
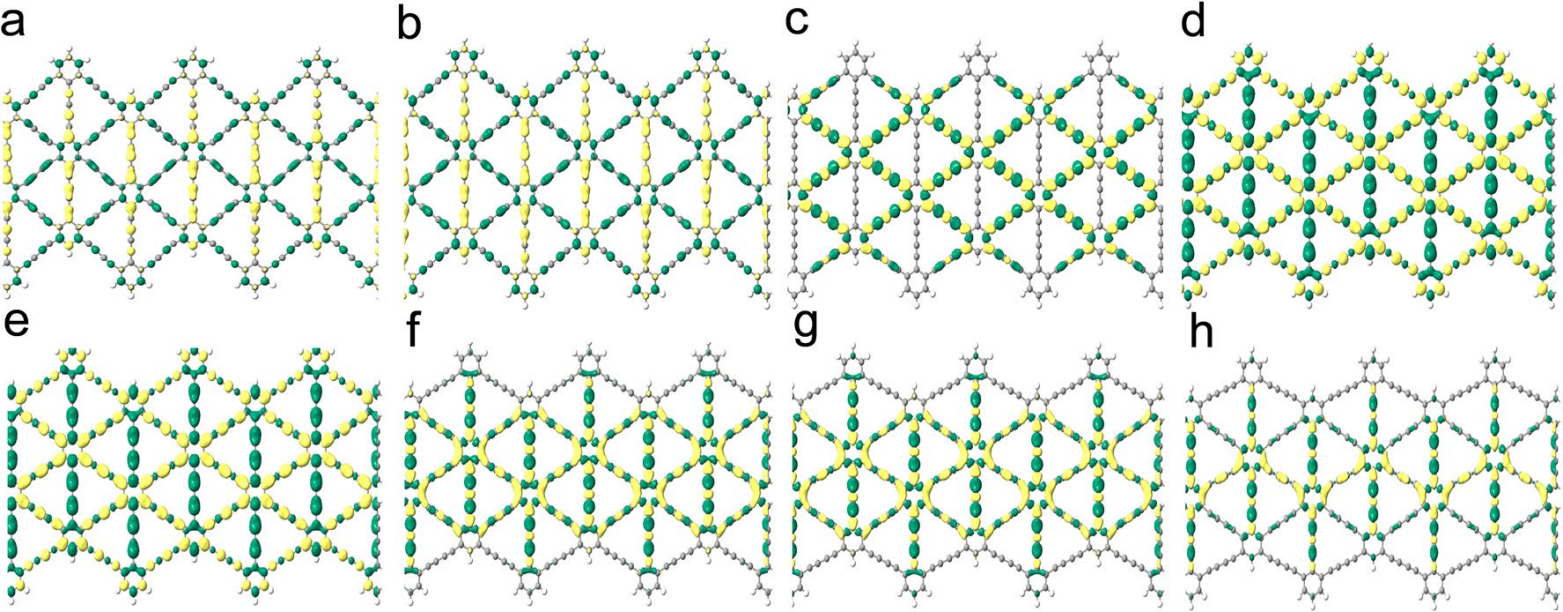
CDD of Z(3)-GDYNR at the gamma point. (**a**) − 3.5 GPa; (**b**) − 2 GPa; (**c**) 0 GPa; (**d**) 0.5 GPa; (**e**) 2 GPa; (**f**) 3.5 GPa; (**g**) 5 GPa; (**h**) 7.5 GPa.

Figures 5 and 6 show the charge difference densities (CDD)of A(3)-GDYNR and Z(3)-GDYNR between CB and VB at the Gamma point, respectively. This diagram visualizes the major contribution of chemical bonds to the change of structural band gap under stress. The yellow equivalent plane represents the part where electrons accumulate, and the green equivalent plane represents the part where electrons are lost. Set the isosurface value to "0.0002".

It can be observed that the CDD of the structure is different when compressive and tensile stress are applied externally. When externally compressed or in the absence of external stress, the charge of A(3)-GDYNR is mainly located at the edge atoms (Fig. 5). However, when tensile stress is applied externally, the CDD of A(3)-GDYNR changes separately. As shown in Fig. 5d–f, the CDD is mainly in the non-edge atoms. Under tensile stress, the change of band gap is mainly caused by the atoms in A(3)-GDYNR. This also indicates that the bandgap variation trend of A(3)-GDYNR under compressive stress is different from that under tensile stress (Fig. 2c). The CDD of Z(3)-GDYNR is also studied. Similar to the charge distribution of A(3)-GDYNR. The non-edge charge distribution is mainly contributed by the atoms at the benzene ring inside the structure. This results in a similar trend of energy band variation between the two structures. The band gap decreases with the increase of stress. In addition, the difference is that the difference in the edge morphology of the two structures leads to the difference in the sub-edge charge distribution. Therefore, the band gap change is not exactly the same. Under the same external stress, the band gap change of Z(3)-GDYNR is not obvious compared with that of A(3)-GDYNR.

**Figure 7:**
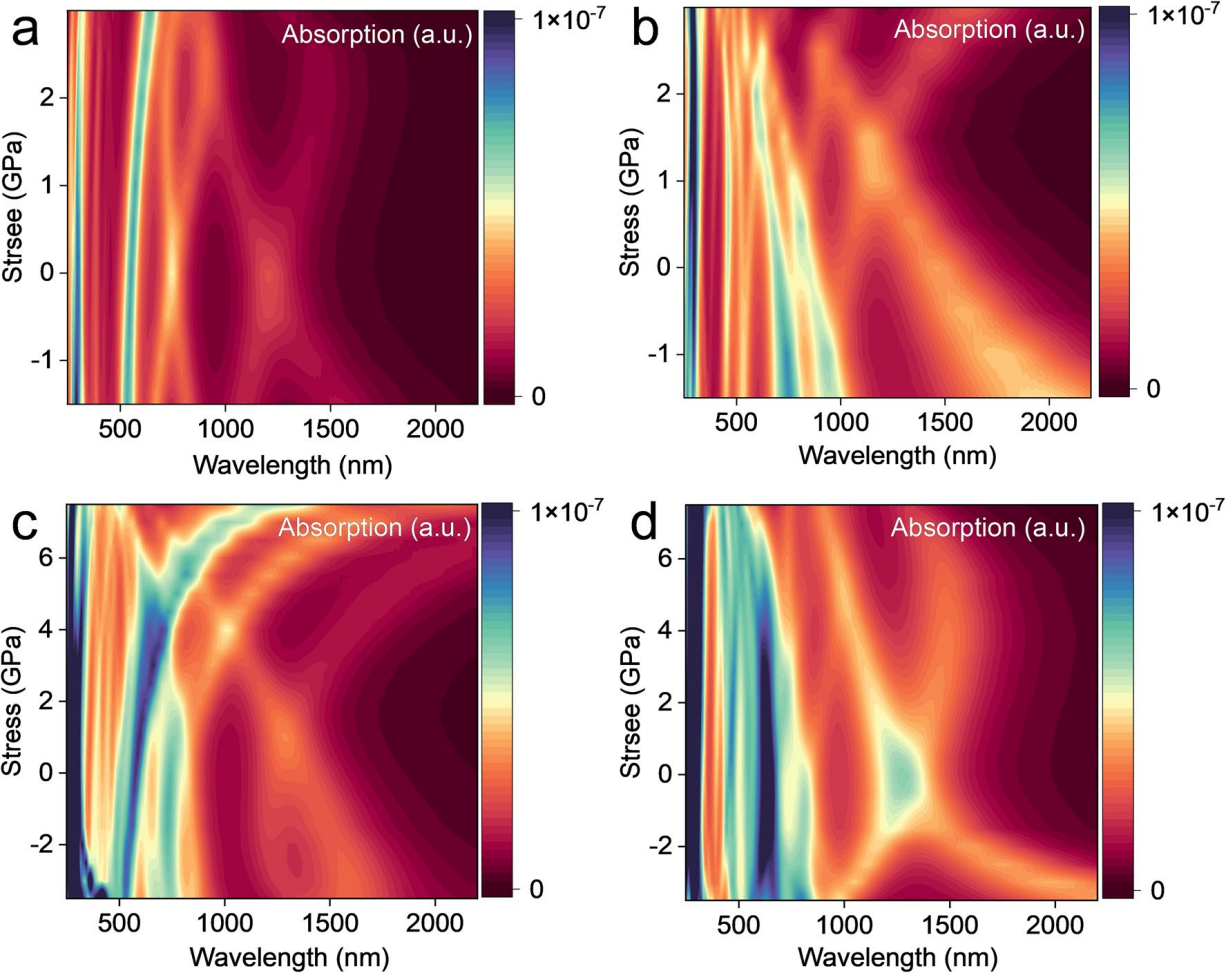
(**a**) Absorption spectrum of the A(3)-GDYNR, under the vertical stress. In plane direction of A(3)-GDYNR; (**b**) vertical the plane direction; (**c**) absorption spectrum of the Z(3)-GDYNR under the vertical stress. In plane direction of Z(3)-GDYNR; (**d**) vertical the plane direction.

In addition, the effects of external stress on the optical properties of A(3)-GDYNR and Z(3)-GDYNR are also studied. Figure 7a, b show the absorption spectra of A(3)-GDYNR in the vertical plane direction and in the in-plane direction with a wavelength of 250–2200 nm under the regulation of vertical stress. Figure 7c, d show the absorption spectra of Z(3)-GDYNR in the vertical plane direction and in the in-plane direction at the wavelength of 250–2200 nm respectively. It was observed that the absorption spectra of these two structures showed obvious anisotropy in two different directions.

Figure S6 shows the UV–Vis spectrum of A(3)-GDYNR and Z(3)-GDYNR at ground state. In the UV–Vis (400–780 nm) range, the multimodal optical absorption coefficients of the two structures showed significant changes with the increase of stress. This is because peaks in the absorption spectrum are caused by transitions between electron orbitals. And the energy difference between the orbitals is fixed. The position of the absorption peak doesn't change. Due to the modulation of the external electric field to the energy band, the transition dipole moment of the electronic transition changes, and the absorption intensity changes.

Specifically, as shown in Fig. 7a, A(3)-GDYNR has weak peaks near 300 nm and 500 nm, and the light absorption coefficient near 500 nm shows an obvious redshift with the increase of stress. With the increase of stress, the absorption coefficient at 300 nm also shows a significant redshift. Different from the light absorption coefficient of about 500 nm. When the applied stress is adjusted at − 1.5 to 1.5 GPa, the light absorption coefficient has a significant enhancement. And then gradually weakens until it disappears with the increase of the applied stress. In Fig. 7b, a significant redshift occurs when the wavelength is around 300 nm. The light absorption peak at 800 nm appears at − 1.5 GPa and disappears at 0 GPa. At 1500–2000 nm, there is an obvious redshift phenomenon. The redshift decreases with the increase of stress and disappears at 2.5 GPa.

In Fig. 7c, at 500 nm, the absorption coefficient shows a significant redshift as the stress increases. At 300 nm, the light absorption coefficient increases significantly when the stress is adjusted from − 3.5 to 2GPa. Unlike 500 nm, the light absorption coefficient at 300 nm gradually decreases until it disappears with the increase of the external compressive stress. In Fig. 7d, the absorption coefficient appears around 300 nm. When the stress is adjusted to 2.5 GPa, the light absorption coefficient gradually increases with the increase of stress. When the stress is − 2 to 2 GPa, a weak peak appears at 600 nm. At 1000–1500 nm, the light absorption coefficient increases with the increase of stress.

**Figure 8:**
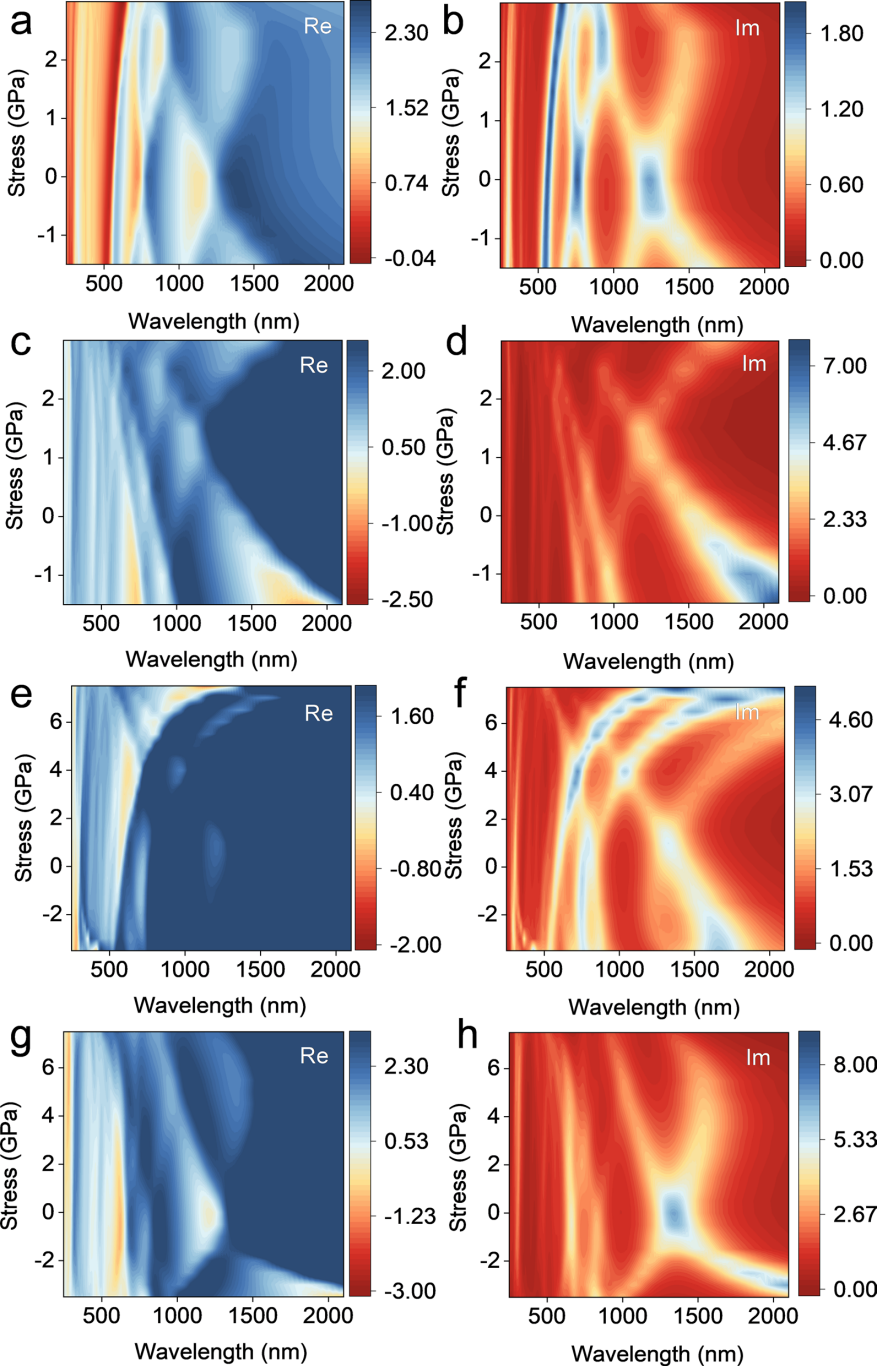
Dielectric constant of A(3)-GDYNR structure when stress is applied vertical the plane direction. (**a, b**) In plane direction; (**c, d**) vertical the plane direction, Dielectric constant of Z(3)-GDYNR structure when stress is applied vertical the plane direction; (**e, f**) in plane direction; (**g, h**) vertical the plane direction.

Since the dielectric constant of a material describes the energy, wavelength or frequency related electrical and optical properties. It is a key factor in device simulation. The imaginary part of the dielectric constant is obtained by calculating the integral of the electron transition from the occupied state to the non-occupied state. Then, the real part of the dielectric constant is obtained by the Krammer-Kronig transformation^60^. The real and imaginary parts of permittivity are important for the analysis of electron transition and light absorption characteristics. The surface plasmon effect is derived from the real part of the dielectric constant^60^. In this work, it is found that the dielectric constants of A(3)-GDYNR and Z(3)-GDYNR perpendicular to the plane direction and in the plane direction are significantly anisotropic under uniaxial tension and compressive stress perpendicular to the plane direction (Fig. 8).

Specifically, in the in-plane direction of A (3)-GDYNR, the real part of the dielectric constant exhibits a weak surface plasmons effect at 629 nm (Fig. 8a). The imaginary part of the dielectric constant peaks at 1200 nm and decreases with the increase of stress (Fig. 8b). The real part of the dielectric constant of A(3)-GDYNR in the vertical plane direction shows an obvious surface plasmon effect at 250–750 nm and 1400–2000 nm. At the wavelength of 1400–2000 nm, A(3)-GDYNR has obvious surface plasmon effect with the increase of compressive stress. However, this phenomenon disappears when external stress is applied (Fig. 8c). The imaginary part is at 2200 nm, and there is a strong peak at − 1.5 GPa, and the peak value decreases with the increase of stress (Fig. 8d).

In the in-plane direction of Z(3)-GDYNR, the real wavelength of the dielectric constant has a plasmon effect in the range of 250–1200 nm. The surface plasmon effect increases with the increase of stress. The imaginary part peaks at 1200 nm (Fig. 8e, f). In the vertical plane direction of Z(3)-GDYNR, the real part of the dielectric constant has a surface plasmon effect at 280 nm, 600 nm and 2300 nm. The surface plasmon effect decreases at 280 nm and 600 nm with the increase of stress wavelength. When the stress is − 3.5 GPa, the imaginary part is negative and there is a surface plasmon effect. When the stress is − 2 to 0 GPa, the imaginary part of the dielectric constant peaks at 1200–4200 nm. And the peak value gradually decreases with the increase of stress (Fig. 8g, h).

In order to observe the effect of stress on materials more accurately, Poisson's ratio is introduced in this work. Poisson's ratio is used to measure the ratio of uniaxial stress to strain of a material under uniaxial tensile and compressive stress. Is the elastic constant that reflects the transverse deformation of the material^25^. Uniaxial tensile stress is applied to A(3)-GDYNR and Z(3)-GDYNR in the Z direction. With the increase of tensile stress, the lattice length of the structure in the Z direction increases, and the lattice length perpendicular to the Z direction decreases. Conversely, uniaxial compressive stress in the Z direction is applied to the material. As the compressive stress increases, the lattice length of the structure decreases in the Z direction and increases in the lattice length perpendicular to the Z direction (Fig. 2c, d).

**Figure 9:**
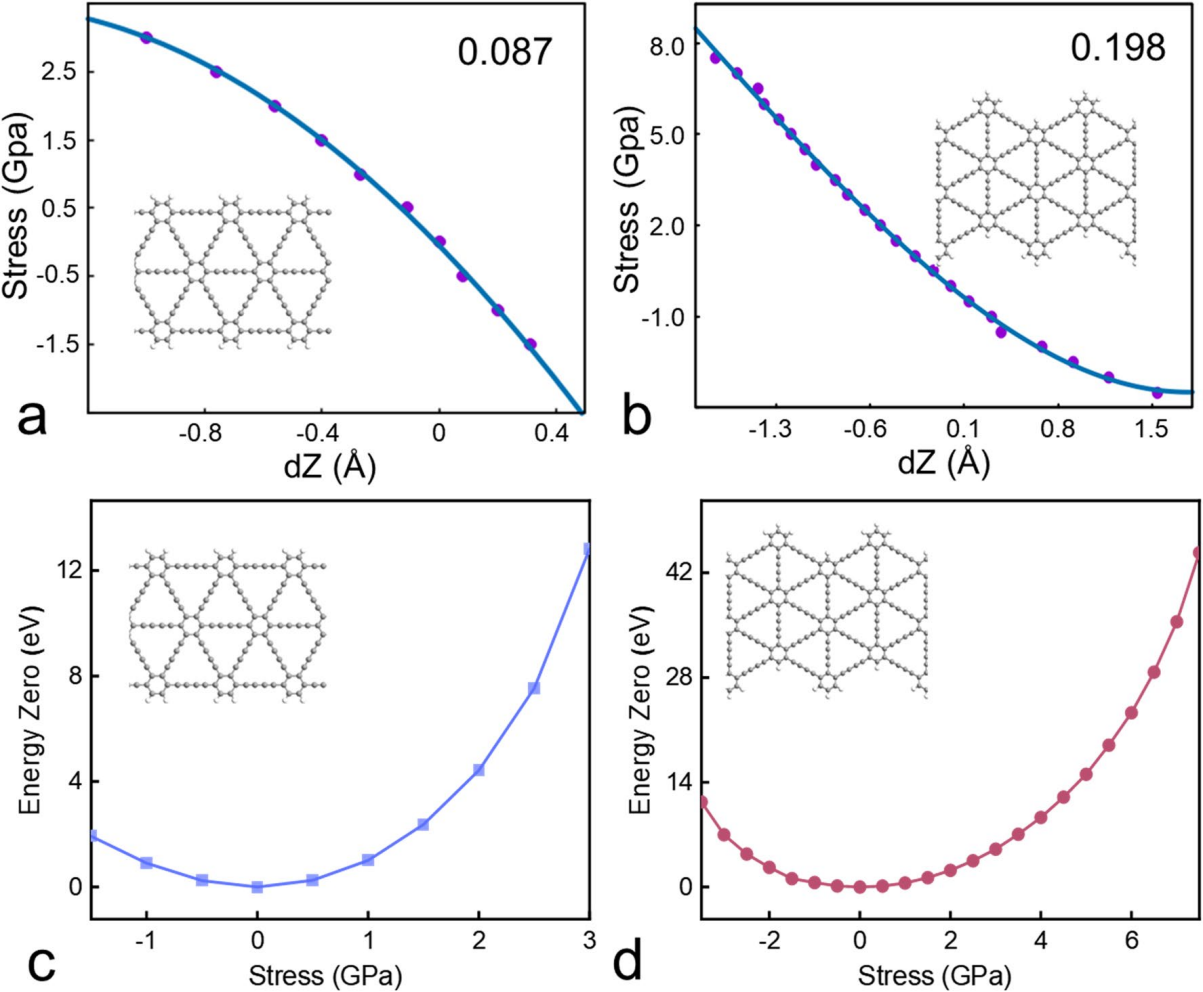
(**a**) Fitting the stress–strain curves of A(3)-GDYNR; (**b**) fitting the stress–strain curves of Z(3)-GDYNR; (**c, d**) ground state energies of A(3)-GDYNR and Z(3)-GDYNR structures when different stress are applied perpendicular to the plane direction.

As shown in Fig. 9a, b, the Poisson ratios of A(3)-GDYNR and Z(3)-GDYNR are calculated to be 0.087 and 0.198, respectively. This also shows that under the same external stress, the degree of deformation affecting A(3)-GDYNR is smaller than that affecting Z(3)-GDYNR. This is because A(3)-GDYNR and Z(3)-GDYNR have different structures. Z(3)-GDYNR has more acetylene chains than A(3)-GDYNR. As the acetylene chain increases, the carbon atoms become closer together in the plane structure and the fracture stress decreases. Resulting in increased structural hardness.

Then, in order to evaluate the stability of the structure. The ground state energies of A(3)-GDYNR and Z(3)-GDYNR are also calculated (Fig. 9c, d). In this work, the energy value under the stress of 0 GPa is taken as the zero point for reference. The ground state energies of A(3)-GDYNR and Z(3)-GDYNR both increase with externally applied stress. The stability decreases with the increase of applied stress. Although the change trend of these two structures is consistent, there are still significant differences. It can be clearly observed from Fig. 9c, d that Z(3)-GDYNR is less stable than A(3)-GDYNR when the same stress is applied externally. This is also consistent with the statement mentioned above. That is, under the same external stress, the deformation degree of A(3)-GDYNR is smaller than that of Z(3)-GDYNR.

## Discussion

In summary, the electronic structure and optical properties of A(3)-GDYNR and Z(3)-GDYNR regulated by uniaxial tensile and compressive stress are theoretically calculated by using the first principles calculation method. Firstly, the influence of external stress on the stability of the two structures is calculated. With the increase of stress, the stability of both structures showed a decreasing trend. AIMD simulations of A(3)-GDYNR and Z(3)-GDYNR under stress induction were performed. The results showed that the A(3)-GDYNR and Z(3)-GDYNR structures maintained well without significant distortion or collapse, and the difference of bond length before and after AIMD simulation was less than 0.06 Å. In addition, the AIMD simulations of A(3)-GDYNR and Z(3)-GDYNR at 300 K and 600 K are also studied. The results show that with the increase of simulated temperature and applied stress, the geometric fluctuation caused by thermal motion increases. In order to further confirm the stability of the structure, the phonon spectra of A(3)-GDYNR and Z(3)-GDYNR are also calculated. There are no virtual frequencies in the phonon spectrum.

Subsequently, it is also found that the applied stress can regulate the electronic properties and band structure of A(3)-GDYNR and Z(3)-GDYNR. In A(3)-GDYNR structure, the bond length at E, B and D also shows a significant increase trend with the increase of applied stress. Because of the change of the interaction force between the atoms connected by the stretched bond, the band gap decreases with the increase of the applied stress. Different from A(3)-GDYNR, the acetylene chain in Z(3)-GDYNR structure is not parallel to the direction of the applied stress, the external stress mainly affects the bond Angle in Z(3)-GDYNR structure, but not the bond length. With the same external stress, the bond length of Z(3)-GDYNR structure increases less than that of A(2)-GDYNR. The band gap decreases with the increase of applied stress. It can be intuitively observed from Fig. 2c–d that the lattice of the structure in the Z direction changes with stress. In addition, in order to obtain the quantitative scaling between the structural band gap and the applied compression, tensile stress. In this paper, the functional relationship between A(3)-GDYNR and Z(3)-GDYNR band gap and applied compressive and tensile stress is studied. The results show that the band gap change of A-GDYNRs is more affected by external forces than that of Z-GDYNRs.

In this work, the effects of uniaxial tensile and compressive stress on the electronic properties of A(3)-GDYNR and Z(3)-GDYNR are studied. The Bloch wave functions and CDD of the two structures are shown in this work. By calculating the effective mass of electrons and holes, it is found that the conduction reasons of A(3)-GDYNR and Z(3)-GDYNR are different under different stress. The electron and hole effective mass of A(3)-GDYNR is smaller than that of Z(3)-GDYNR. Poisson ratios of A(3)-GDYNR and Z(3)-GDYNR are calculated to be 0.087 and 0.198, respectively. This shows that under the same external stress, the degree of deformation affecting A(3)-GDYNR is smaller than that affecting Z(3)-GDYNR.

In addition, the application of stress can distort the lattice of materials, affect the band structure, and regulate the band gap size of the materials. It also affects the optical properties of GDYNRs. In this work, the wavelength range of the surface plasma of A(3)-GDYNR and Z(3)-GDYNR in different directions is adjusted by means of applied stress. Therefore, this work provides an efficient method for the modulation of electronic and optical properties of A(3)-GDYNR and Z(3)-GDYNR, including edge morphology and stress. All of these methods can adjust the electronic structure of the material without introducing other external factors. GDYNRs has great application potential in novel stress-adjustable nanodevices.

### Supplementary Information

Supplementary Information.

## Data Availability

Data is provided within the manuscript or supplementary information files.

## References

[1] Breeze JD, Salvadori E, Sathian J, Alford NM, Kay WM (2018). Continuous-wave room-temperature diamond maser. Nature.

[2] Luo K, Liu B, Hu WT, Dong X, Wang YB, Huang Q, Gao YF, Sun L, Zhao ZS, Wu YJ, Zhang Y, Ma MD, Zhou XF, He JL, Yu DL, Lu ZY, Xu B, Tian YJ (2022). Coherent interfaces govern direct transformation from graphite to diamond. Nature.

[3] Thess A, Lee R, Nikolaev P, Dai HJ, Petit P, Robert J, Xu CH, Lee YH, Kim SG, Rinzler AG, Colbert DT, Scuseria GE, Tomanek D, Fischer JE, Smalley RE (1996). Crystalline ropes of metallic carbon nanotubes. Science.

[4] Liu C, Fan YY, Liu M, Cong HT, Cheng HM, Dresselhaus MS (1999). Hydrogen storage in single-walled carbon nanotubes at room temperature. Science.

[5] Laplaze D, Bernier P, Maser WK, Flamant G, Guillard T (1998). Carbon nanotubes: The solar approach. Carbon.

[6] Yang F, Wang X, Zhang D, Yang J, Luo D, Xu Z, Wei J, Wang JQ, Xu Z, Peng F, Lee X, Lee L, Lee Y, Lee M, Bai XD, Ding F, Lee Y (2014). Chirality-specific growth of single-walled carbon nanotubes on solid alloy catalysts. Nature.

[7] Dubey R, Dutta D, Sarkar A, Chattopadhyay P (2021). Functionalized carbon nanotubes: Synthesis, properties and applications in water purification, drug delivery, and material and biomedical sciences. Nanoscale Adv..

[8] Kroto HW, Heath JR, O’Brien SC, Curl RF, Smalley RE (1985). C60: Buckminsterfullerene. Nature.

[9] Hou LX, Cui XP, Guan B, Wang SZ, Li R, Liu YQ, Zhu DB, Zheng J (2022). Synthesis of a monolayer fullerene network. Nature.

[10] Xie SY, Gao F, Lu X, Huang RB, Wang CR, Liu ML, Deng SL, Zheng LS (2004). Capturing the labile fullerene(50) as C50Cl10. Science.

[11] Krätschmer W, Lamb LD, Fostiropoulos K, Donald HR (1990). Solid C60: A new form of carbon. Nature.

[12] Novoselov KS, Geim AK, Morozov SV, Jiang D, Zhang Y, Dubonos SV, Grigorieva IV, Firsov AA (2004). Electric field effect in atomically thin carbon films. Science.

[13] Novoselov KS, Geim AK, Morozov SV, Jiang D, Zhang Y, Dubonos SV, Grigorieva IV, Firsov AA (2005). Two-dimensional gas of massless Dirac fermions in graphene. Nature.

[14] Katsnelson MI, Novoselov KS, Geim AK (2006). Chiral tunnelling and the Klein paradox in graphene. Nat. Phys..

[15] Moreau N, Brun B, Somanchi S, Watanabe K, Taniguchi T, Stampfer C, Haackens B (2021). Upstream modes and antidots poison graphene quantum Hall effect. Nat. Commun..

[16] Yin XP, Wang HJ, Tang SF, Liu XL, Shu M, Si R (2018). Engineering the coordination environment of single-atom platinum anchored on graphdiyne for optimizing electrocatalytic hydrogen evolution. Angew. Chem. Int. Ed..

[17] Serafifini P, Milani A, Tommasini M, Bottani CE, Casari CS (2021). Topology-dependent conjugation effects in graphdiyne molecular fragments. Carbon.

[18] Guo J, Guo M, Wang F, Jin WY, Chen CY, Liu HB, Li YL (2020). Graphdiyne: Structure of fluorescent quantum dots. Angew. Chem. Int. Ed..

[19] He F, Li YL (2023). Advances on theory and experiments of the energy applications in graphdiyne. CCS Chem..

[20] Steven W, Dieter BB, Markus JB (2012). Extended graphynes: Simple scaling laws for stiffness, strength and fracture. Nanoscale.

[21] Fang Y, Liu L, Qi L, Xue YR, Li YL (2022). 2D graphdiyne: An emerging carbon material. Chem. Soc. Rev..

[22] Miao WJ, Wang L, Mu XJ, Wang JG (2021). The magical photoelectric and optoelectronic properties of graphene nanoribbons and their applications. J. Mater. Chem. C.

[23] Serafini P, Milani A, Tommasini M, Castiglioni C, Cassari CS (2020). Raman and IR spectra of graphdiyne nanoribbons. Phys. Rev. Mater..

[24] Bhuvaneswari R, Nagarajan V, Chandiramouli R (2020). Explosive vapor detection using novel graphdiyne nanoribbons: A first-principles investigation. Struct. Chem..

[25] Long MQ, Tang L, Wang D, Li YL, Shuai ZG (2011). Electronic structure and carrier mobility in graphdiyne sheet and nanoribbons: Theoretical predictions. ACS Nano.

[26] Song, J. Z., Cao, Y., Dong, J. & Sun, M. T. Superior thermoelectric properties of twist‐angle superlattice borophene induced by interlayer electrons transport. *Small* 2301348 (2023). 10.1002/smll.202301348 36919623

[27] Wan YC, Xiong SY, Ouyang B, Niu ZH, Ni YX, Zhao Y, Zhang XH (2019). Thermal transport engineering in graphdiyne and graphdiyne nanoribbons. ACS omega.

[28] Song JZ, Zhang ZY, Feng NX, Wang JG (2021). Electric field induced twisted bilayer graphene infrared plasmon spectrum. Nanomaterials.

[29] Zhou W, Shen H, Zeng Y, Yi YP, Zuo ZC, Li JY (2020). Controllable synthesis of graphdiyne nanoribbons. Angew. Chem. Int. Ed..

[30] Gog H, Li WF, Fang C, Koster RS, Dijkstra M, Huis M (2019). Thermal stability and electronic and magnetic properties of atomically thin 2D transition metal oxides. NPJ 2D Mater..

[31] Kim AY, Changyub N, Lim A (2024). Crystal structures, phase transitions, thermodynamics, and molecular dynamics of organic–inorganic hybrid crystal [NH(CH_3_)_3_]_2_ZnCl_4_. Sci. Rep..

[32] Li L, Bai H, Li Y, Huang YH (2019). The electronic properties and magnetic states of edge-modified γ-graphdiyne nanoribbons. Comput. Mater. Sci..

[33] Hu G, He J, Chen J, Li JY (2023). Synthesis of a wheel-shaped nanographdiyne. J. Am. Chem. Soc..

[34] Rodrigues DCM, Lage LL, Venezuela P, Latge A (2022). Exploring the enhancement of the thermoelectric properties of bilayer graphyne nanoribbons. Phys. Chem. Chem. Phys..

[35] Cui C, Ouyang T, Tang C, He CY, Li J, Zhang CX, Zhong ZX (2021). Bayesian optimization-based design of defect gamma-graphyne nanoribbons with high thermoelectric conversion efficiency. Carbon.

[36] Tan X, Xu X, Ding L, He YL (2021). Linear and nonlinear thermal spin transport properties of zigzag α-graphyne nanoribbons with sp^2^–sp^3^ edges. Chem. Phys. Lett..

[37] Bai HC, Zhu Y, Qiao WY, Huang YH (2011). Structures, stabilities and electronic properties of graphdiyne nanoribbons. RSC Adv..

[38] Kang J, Wei Z, Li J (2018). Graphyne and its family: Recent theoretical advances. ACS Appl. Mater. Interfaces.

[39] Fan J, Song JZ, Cheng Y, Sun M (2021). Pressure-dependent interfacial charge transfer excitons in WSe_2_–MoSe_2_ heterostructures in near infrared region. Results Phys..

[40] Li G, Li YL, Liu H, Guo YB, Li JY, Zhu DB (2010). Architecture of graphdiyne nanoscale films. Chem. Commun..

[41] Bissett MA, Konabe S, Okada S, Tsuji M, Ago H (2013). Enhanced chemical reactivity of graphene induced by mechanical strain. ACS Nano.

[42] Miao WJ, Sheng H, Wang JG (2023). Vertical stress induced anomalous spectral shift of 13.17° Moiré superlattice in twist bilayer graphene. Molecules.

[43] Xin C, Liao YM, Peng QJ, Yang YD, Cheng C, Zhang WQ, Fang PL, Chen C, Miao L, Jiang JJ (2015). Contrastive band gap engineering of strained graphyne nanoribbons with armchair and zigzag edges. RSC Adv..

[44] Nayebi P, Shamshirsaz M (2020). Effect of vacancy defects on transport properties of α-armchair graphyne nanoribbons. Eur. Phys. J. B.

[45] Song L, Cao H, Chai X, Chen X, Ye Z, Zhou Y, Huang X, Zhu HY, Zheng XH (2020). Highly spin polarized transport in gamma—zigzag graphyne nanoribbon junctions. Physica E.

[46] Miao WJ, Wang L, Wang JG, Sun MT (2023). Simulation studies on external electric field-dependent anisotropy of two-dimensional fullerene nanomaterials qTP C60 and qHP C60: Implications for photoelectric nanodevices. ACS Appl. Nano Mater..

[47] Zheng Q, Luo G, Liu Q, Liu R, Quhe R, Zheng JX, Tang KC, Gao ZX, Nagase S, Lu J (2012). Structural and electronic properties of bilayer and trilayer graphdiyne. Nanoscale.

[48] Serlin M, Tschirhart CL, Polshyn H, Zhang Y, Zhu J, Watanabe K, Taniguchi T, Balents L, Young AF (2020). Intrinsic quantized anomalous Hall effect in a moiré heterostructure. Science.

[49] Chen C, Li J, Sheng XL (2017). Graphdiyne nanoribbons with open hexagonal rings: Existence of topological unprotected edge states. Phys. Lett. A.

[50] Gao X, Liu H, Wang D, Zhang J (2019). Graphdiyne: Synthesis, properties, and applications. Chem. Soc. Rev..

[51] Lin ZZ, Wei Q, Zhu X (2014). Modulating the electronic properties of graphdiyne nanoribbons. Carbon.

[52] Xi J, Nakamura Y, Nakamura Y, Zhao T, Wang D, Shuai ZG (2018). Theoretical studies on the deformation potential, electron-phonon coupling, and carrier transports of layered systems. Acta Phys. Chim. Sin..

[53] Yang S, Chen Y, Jiang C (2021). Strain engineering of two-dimensional materials: Methods, properties, and applications. InfoMat.

[54] Pei Y (2012). Mechanical properties of graphdiyne sheet. Physica B Condens. Matter.

[55] Xiao KL, Li JF, Wu XQ, Liu HB, Huang CG, Li YL (2019). Nanoindentation of thin graphdiyne films: Experiments and molecular dynamics simulation. Carbon.

[56] Yue Q, Chang SL, Kang J, Qin SQ, Li JB (2013). Mechanical and electronic properties of graphyne and its family under elastic strain: Theoretical predictions. J. Phys. Chem. C.

[57] Smidstrup S, Markussen T, Vancraeyveld P, Wellendorff J, Schneider J, Gunst T, Verstichel B, Stradi D, Khomyakov PA, Vej-Hansen UG, Lee M, Chill ST, Rasmussen F, Penazzi G, Corsetti F, Ojanperä A, Jensen K, Palsgaard MLN, Martinez U, Blom A, Brandbyge M, Stokbro K (2019). QuantumATK: an integrated platform of electronic and atomic-scale modelling tools. J. Phys. Condens. Matter..

[58] Setten MJ, Giantomassi M, Bousquet E, Verstraete ML, Hamann DR, Gonze X, Rignanese GM (2018). The PseudoDojo: Training and grading a 85 element optimized norm-conserving pseudopotential table. Comput. Phys. Commun..

[59] Grimme S, Antony J, Ehrlich S, Krieg H (2010). A consistent and accurate ab initio parametrization of density functional dispersion correction (DFT-D) for the 94 elements H-Pu. J. Chem. Phys..

[60] Mohamad A, Mahmoud J, Milad A, Maryam J (2015). Optical properties of two-dimensional zigzag and armchair graphyne nanoribbon semiconductor. Spectrochim. Acta A.

