# Supplementary figures and images for: Exploring the impact of stress on the electronic structure and optical properties of graphdiyne nanoribbons for advanced optoelectronic applications
Source: Sci Rep. 2024 Mar 13;14:6051. doi: 10.1038/s41598-024-56380-z (PMC10937923; doi:10.1038/s41598-024-56380-z)

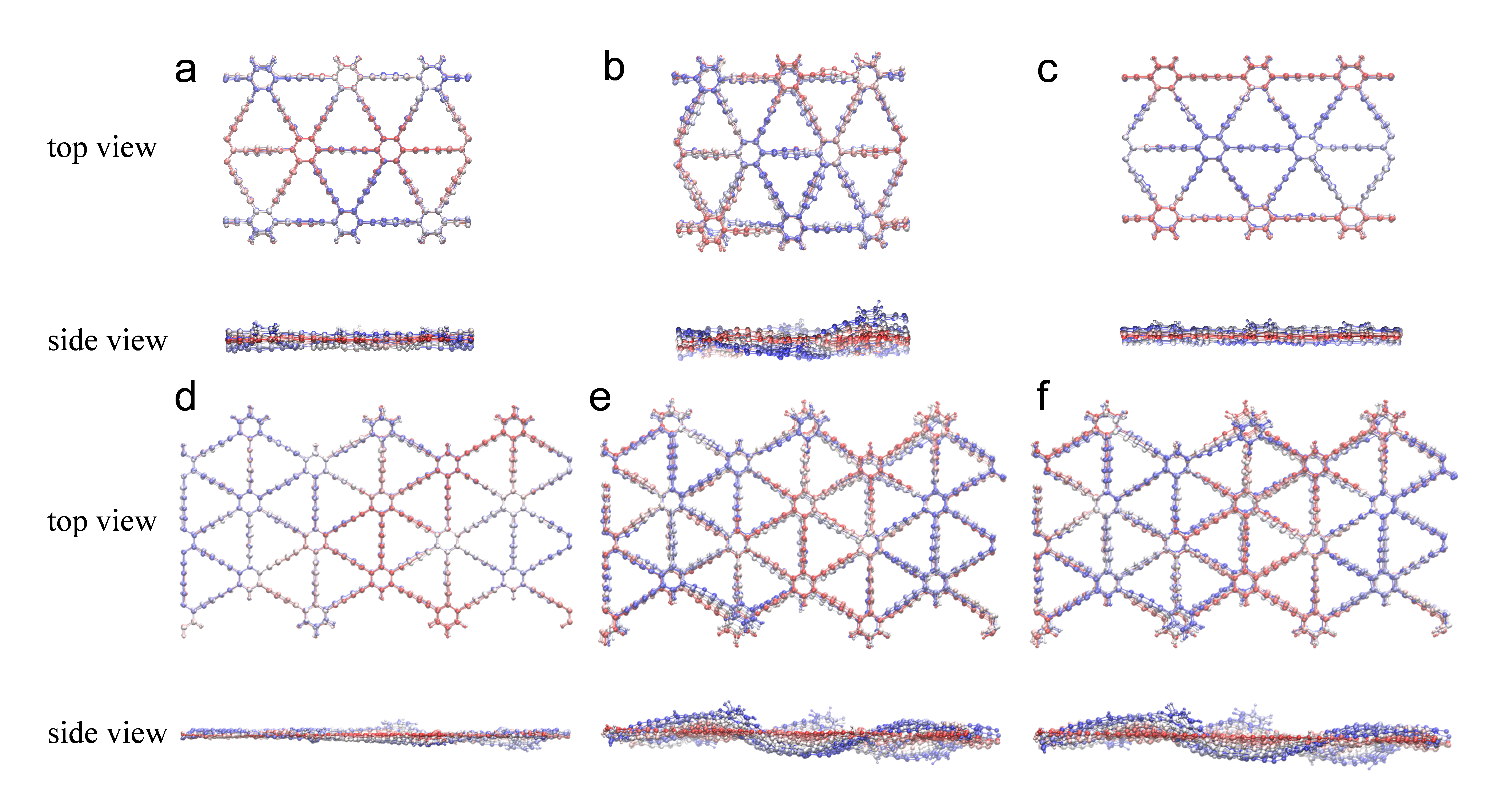

Supplement: Supplementary file 1 — Supplementary Information. [file 41598_2024_56380_MOESM1_ESM.zip › ╓o│┼╨┼╧ó═╝╞1⁄4/Figure S1.png]

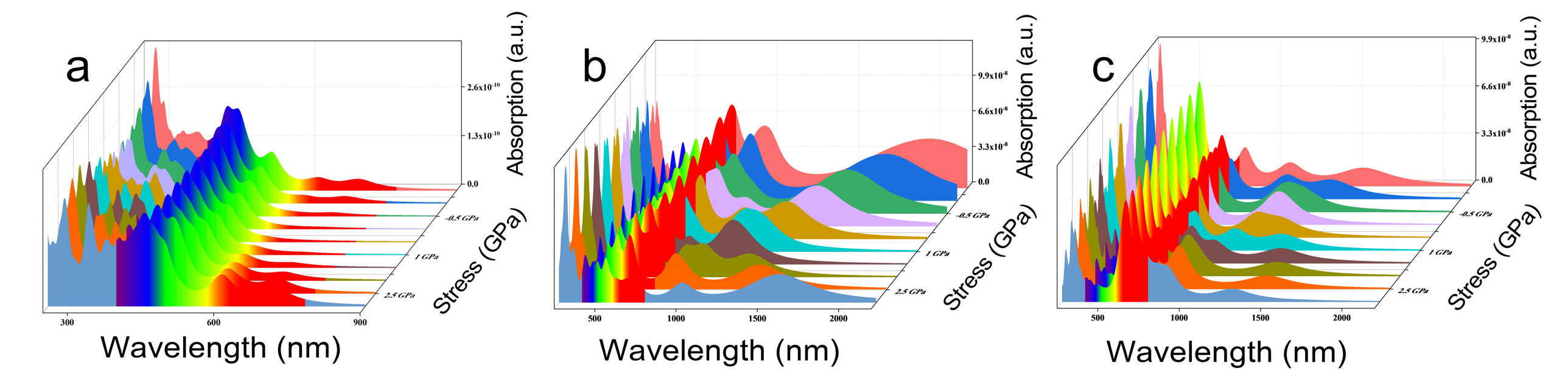

Supplement: Supplementary file 1 — Supplementary Information. [file 41598_2024_56380_MOESM1_ESM.zip › ╓o│┼╨┼╧ó═╝╞1⁄4/Figure S10.png]

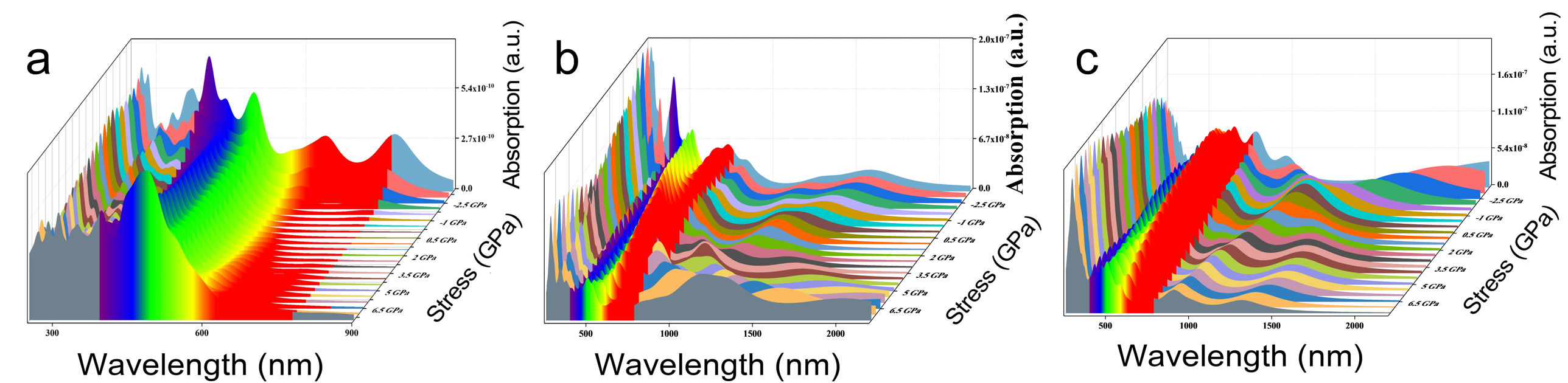

Supplement: Supplementary file 1 — Supplementary Information. [file 41598_2024_56380_MOESM1_ESM.zip › ╓o│┼╨┼╧ó═╝╞1⁄4/Figure S11.png]

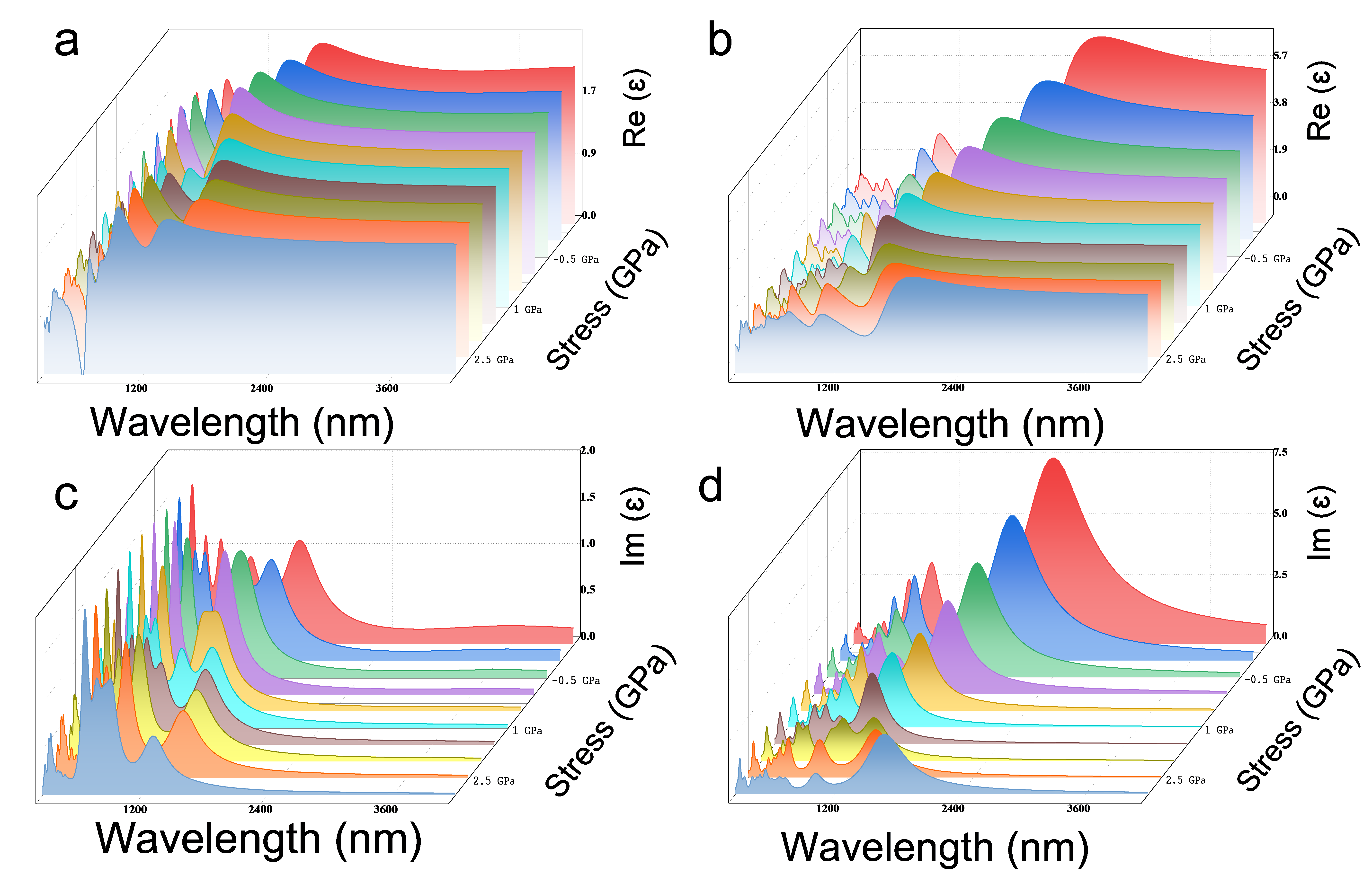

Supplement: Supplementary file 1 — Supplementary Information. [file 41598_2024_56380_MOESM1_ESM.zip › ╓o│┼╨┼╧ó═╝╞1⁄4/Figure S12.png]

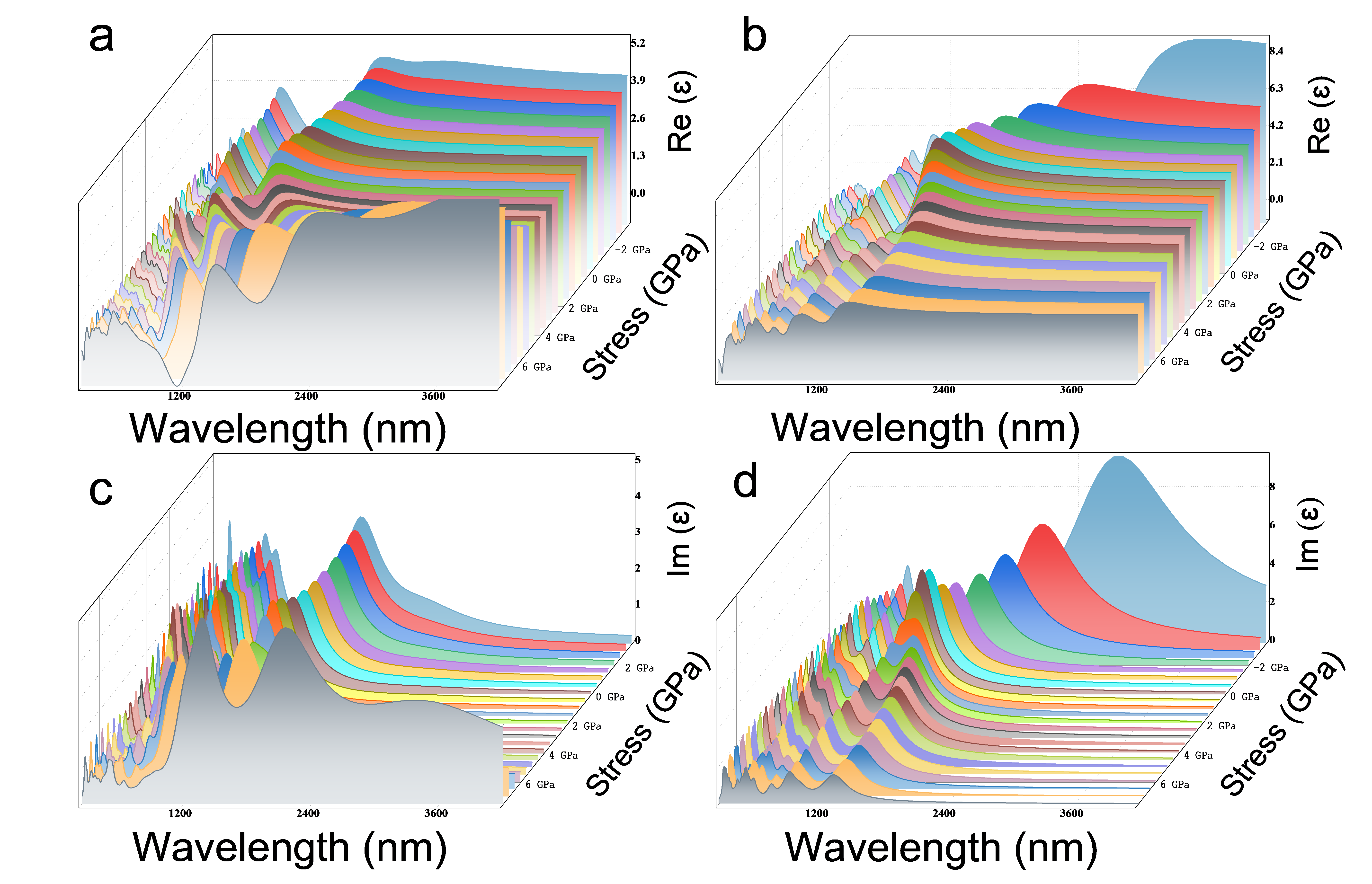

Supplement: Supplementary file 1 — Supplementary Information. [file 41598_2024_56380_MOESM1_ESM.zip › ╓o│┼╨┼╧ó═╝╞1⁄4/Figure S13.png]

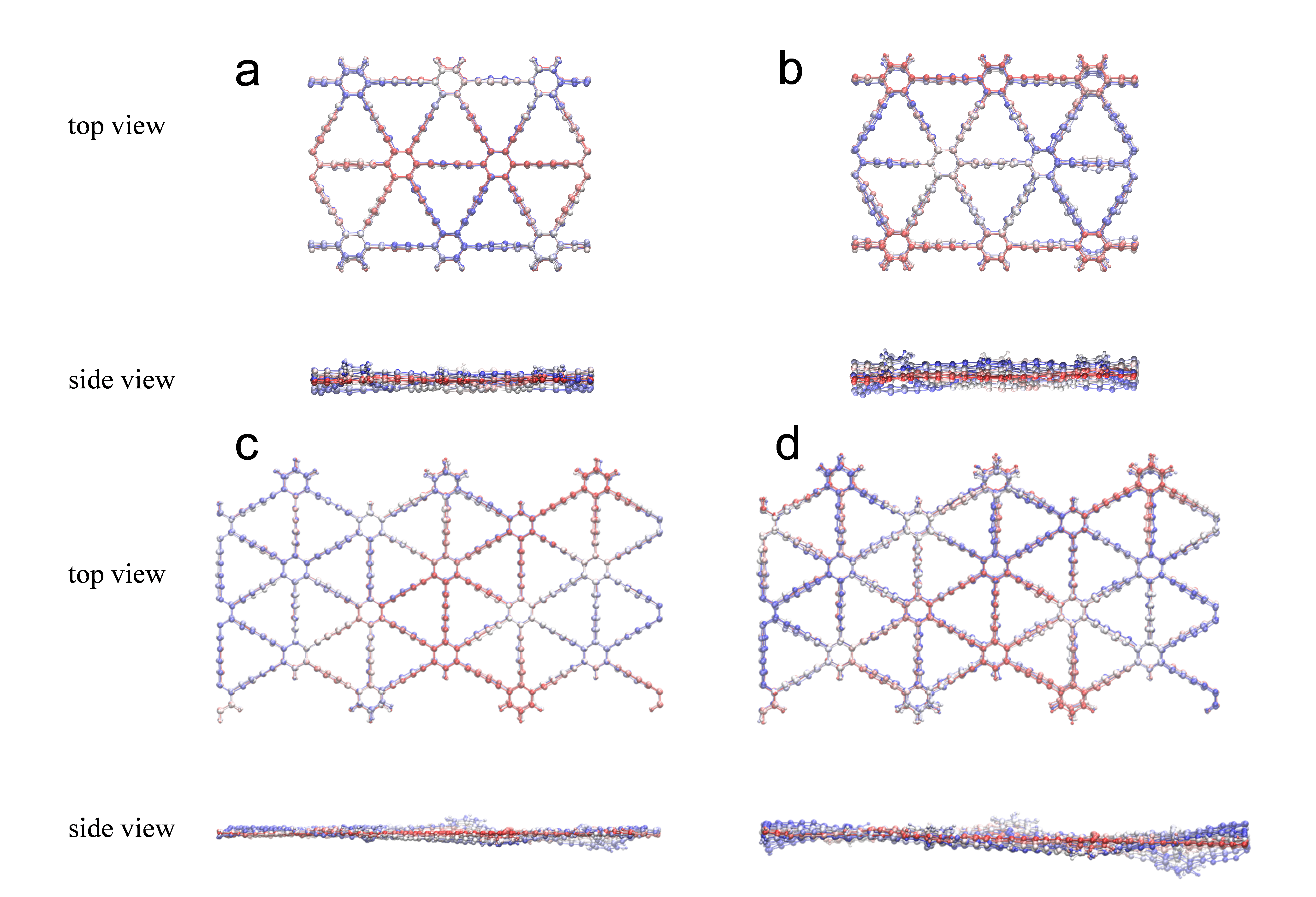

Supplement: Supplementary file 1 — Supplementary Information. [file 41598_2024_56380_MOESM1_ESM.zip › ╓o│┼╨┼╧ó═╝╞1⁄4/Figure S2.png]

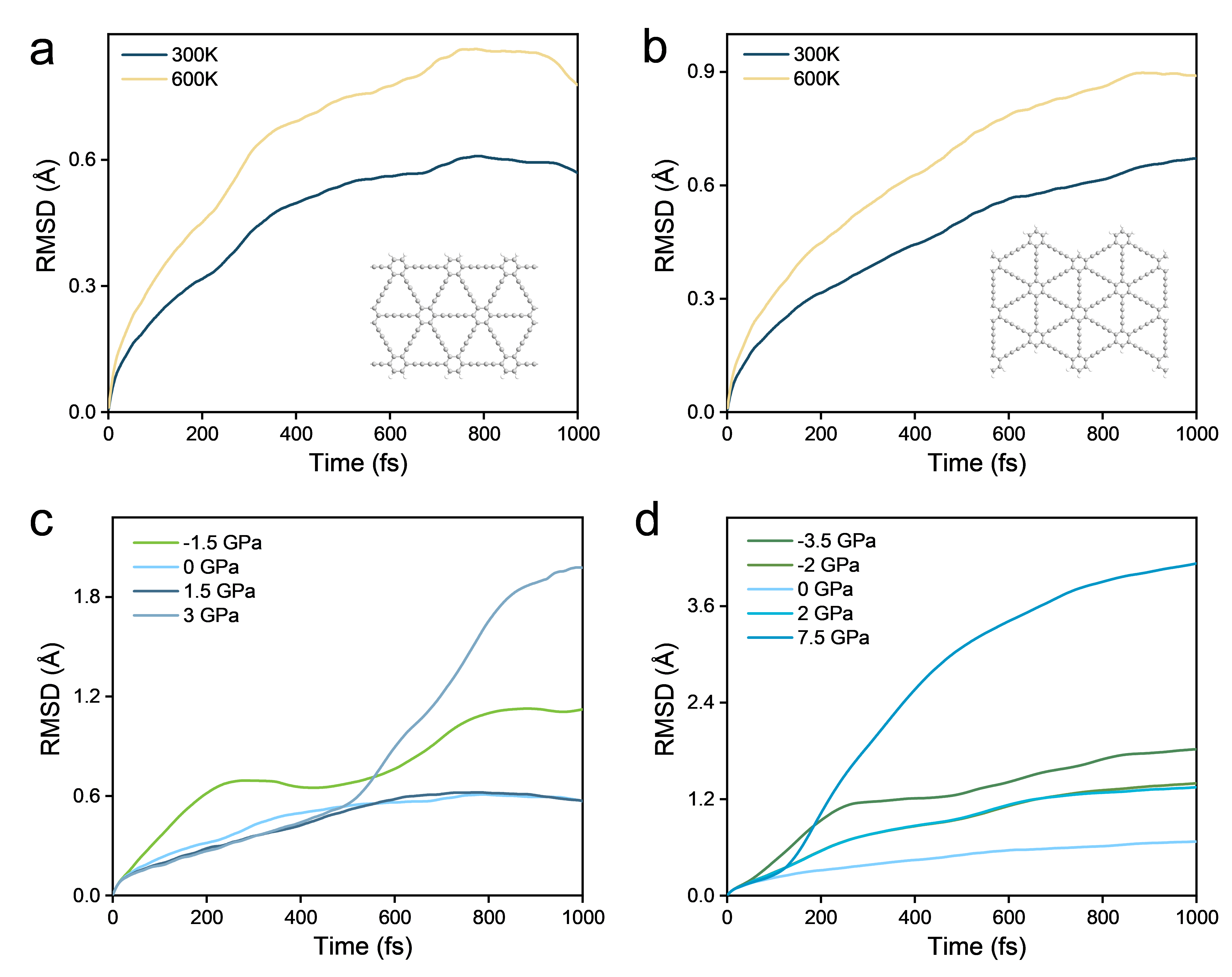

Supplement: Supplementary file 1 — Supplementary Information. [file 41598_2024_56380_MOESM1_ESM.zip › ╓o│┼╨┼╧ó═╝╞1⁄4/Figure S3.png]

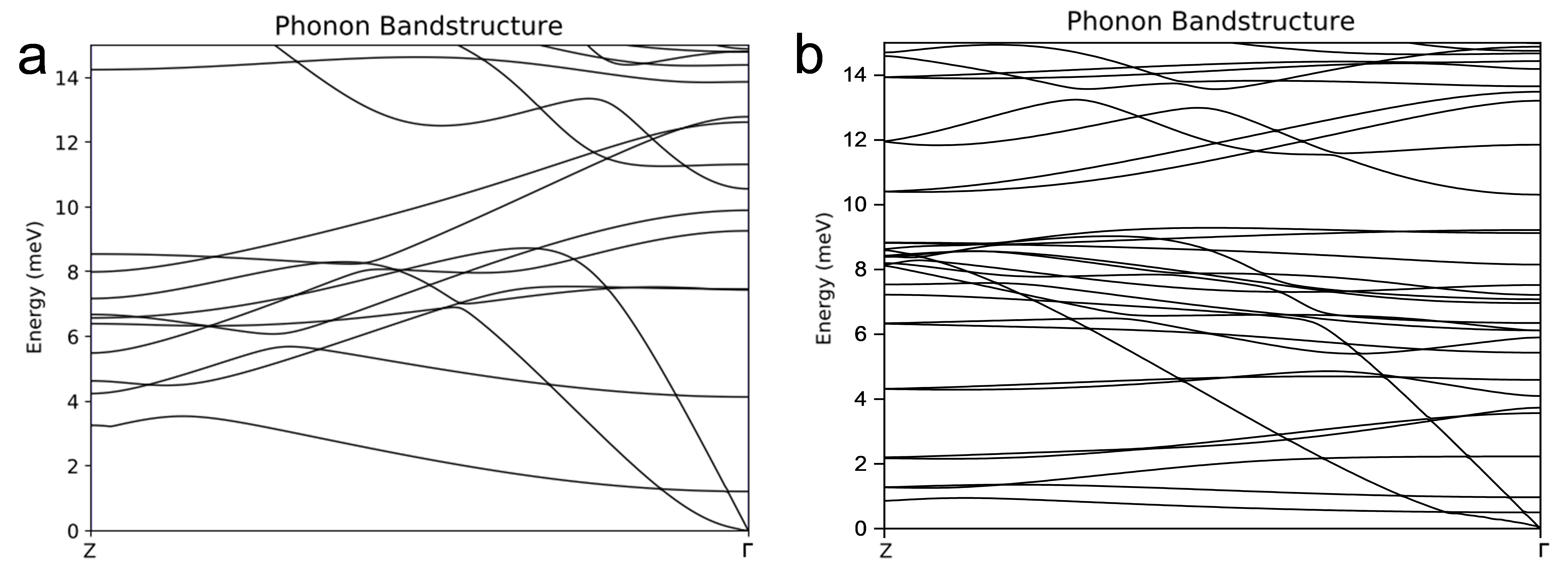

Supplement: Supplementary file 1 — Supplementary Information. [file 41598_2024_56380_MOESM1_ESM.zip › ╓o│┼╨┼╧ó═╝╞1⁄4/Figure S4.png]

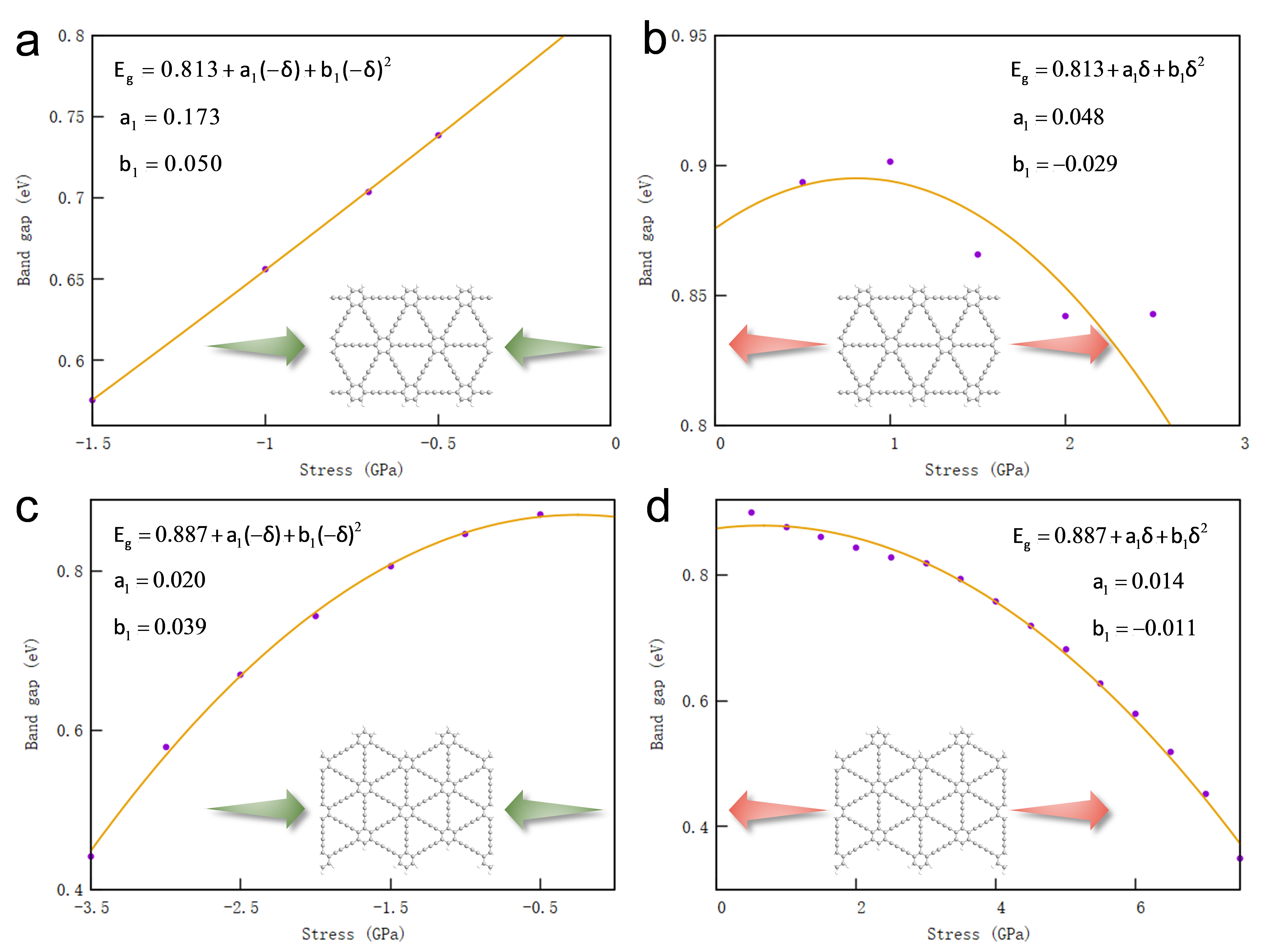

Supplement: Supplementary file 1 — Supplementary Information. [file 41598_2024_56380_MOESM1_ESM.zip › ╓o│┼╨┼╧ó═╝╞1⁄4/Figure S5.png]

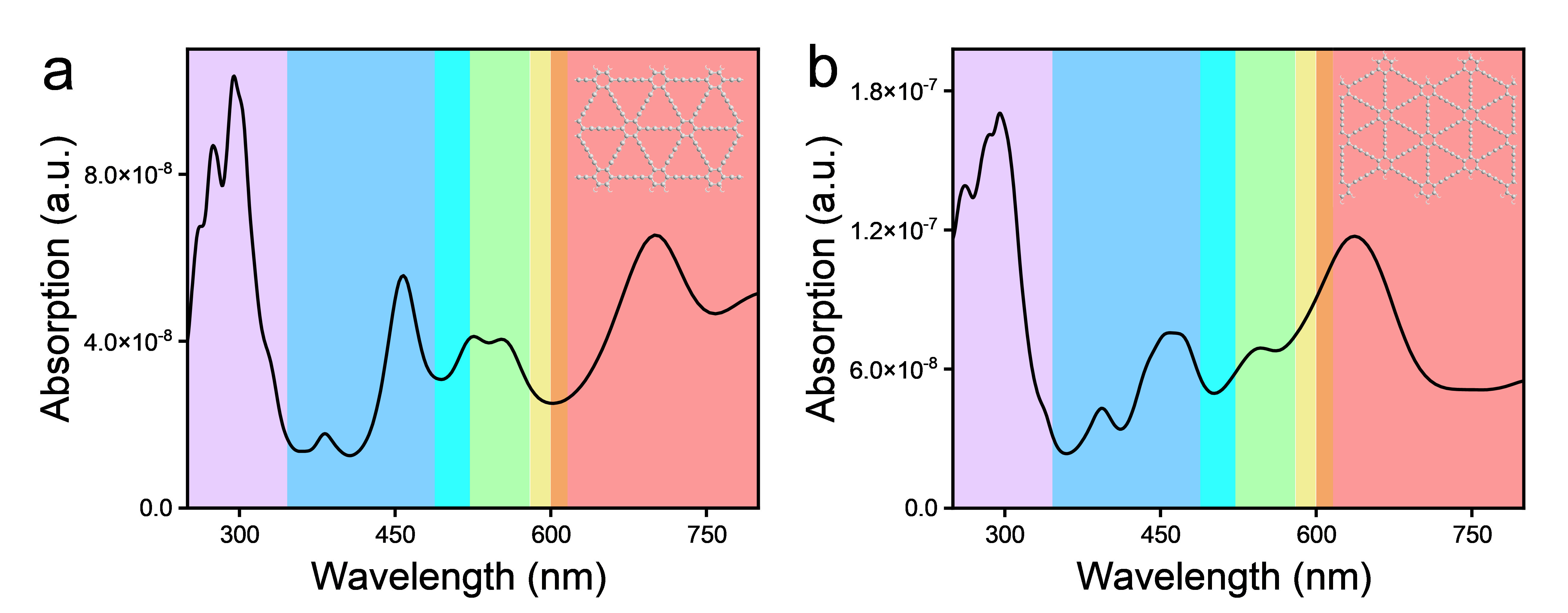

Supplement: Supplementary file 1 — Supplementary Information. [file 41598_2024_56380_MOESM1_ESM.zip › ╓o│┼╨┼╧ó═╝╞1⁄4/Figure S6.png]

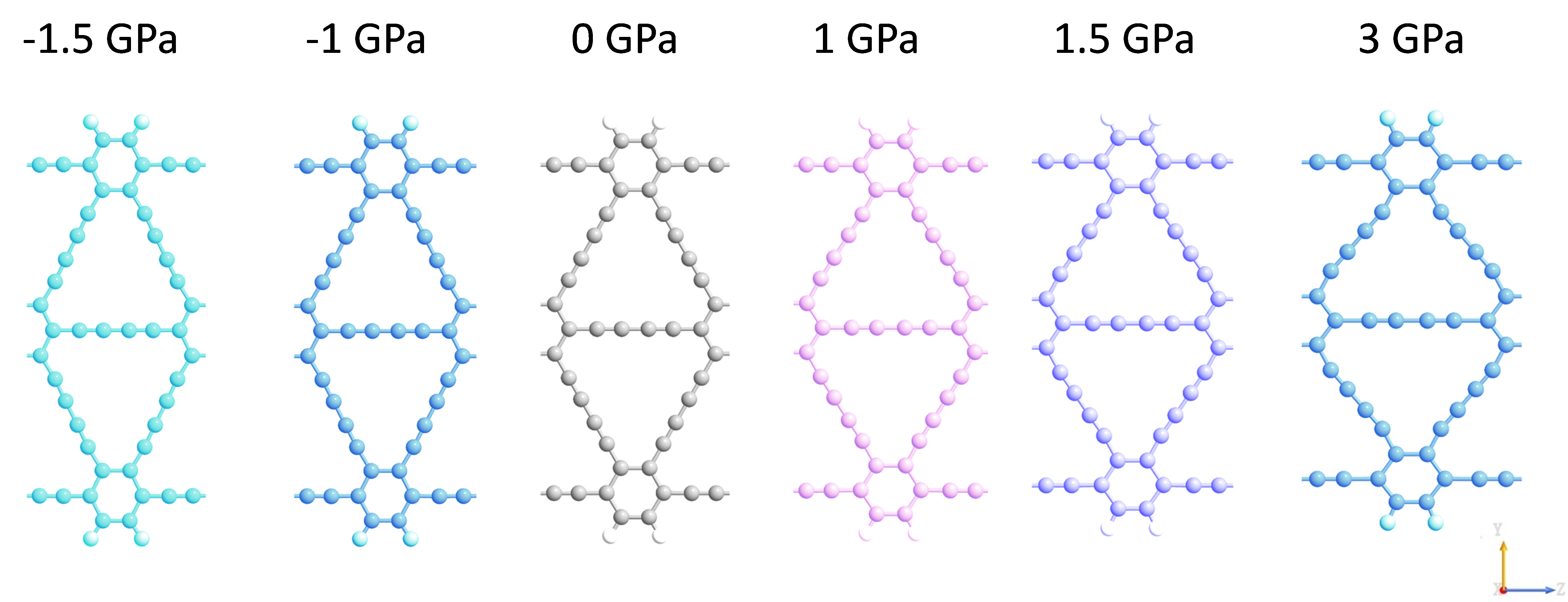

Supplement: Supplementary file 1 — Supplementary Information. [file 41598_2024_56380_MOESM1_ESM.zip › ╓o│┼╨┼╧ó═╝╞1⁄4/Figure S7.png]

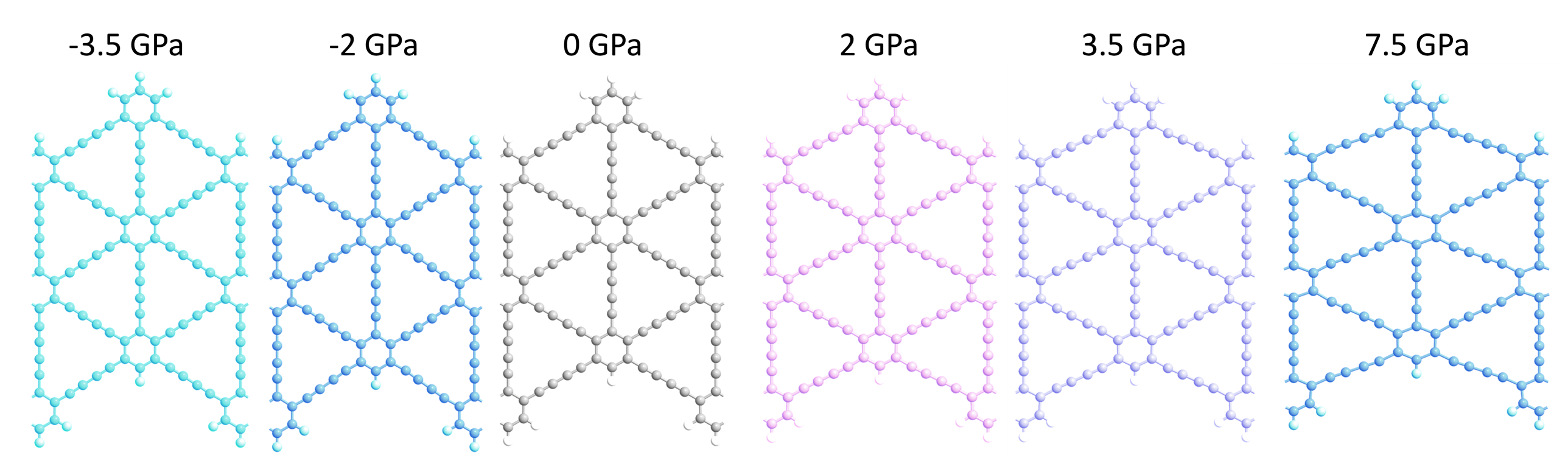

Supplement: Supplementary file 1 — Supplementary Information. [file 41598_2024_56380_MOESM1_ESM.zip › ╓o│┼╨┼╧ó═╝╞1⁄4/Figure S8.png]

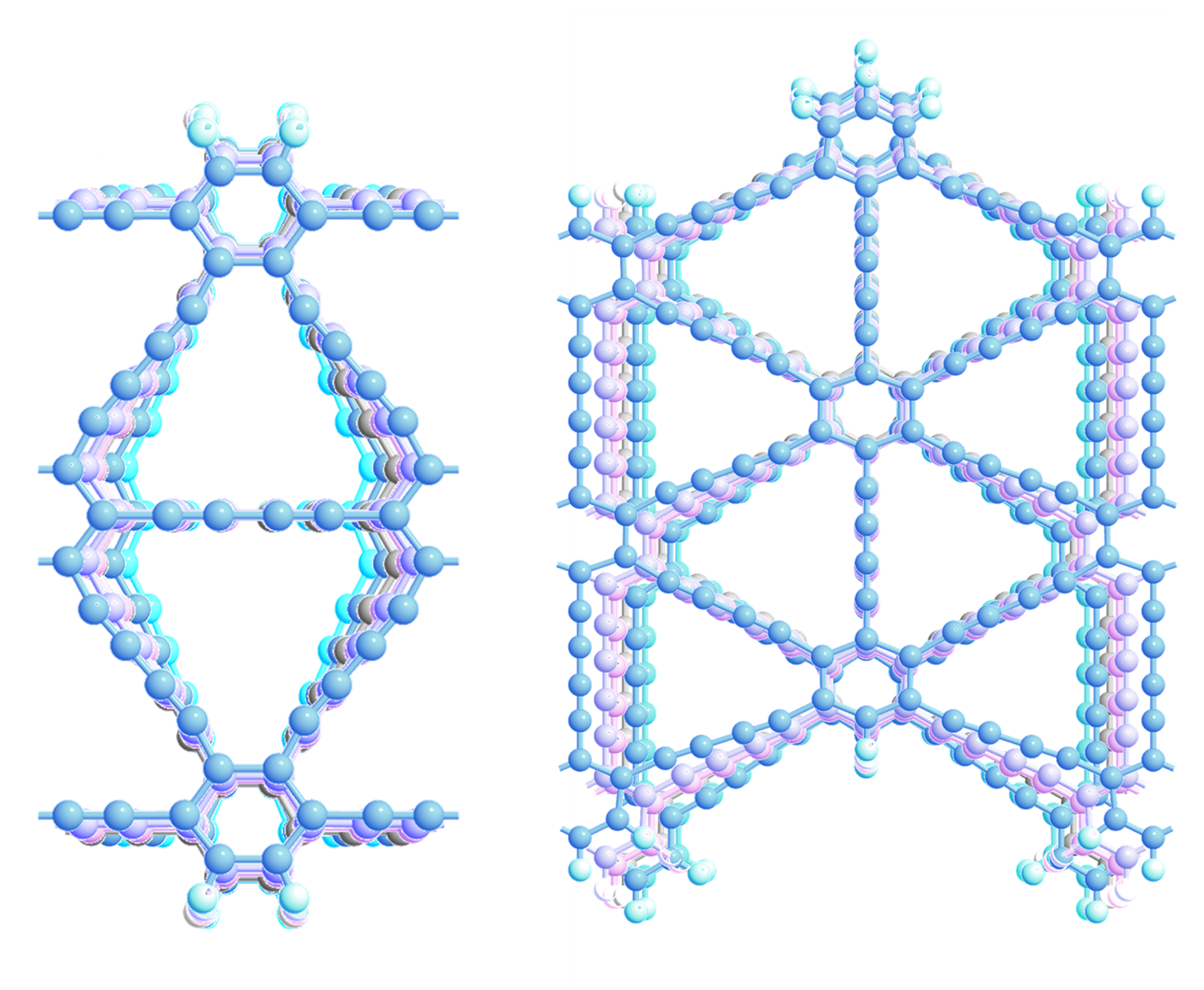

Supplement: Supplementary file 1 — Supplementary Information. [file 41598_2024_56380_MOESM1_ESM.zip › ╓o│┼╨┼╧ó═╝╞1⁄4/Figure S9.png]

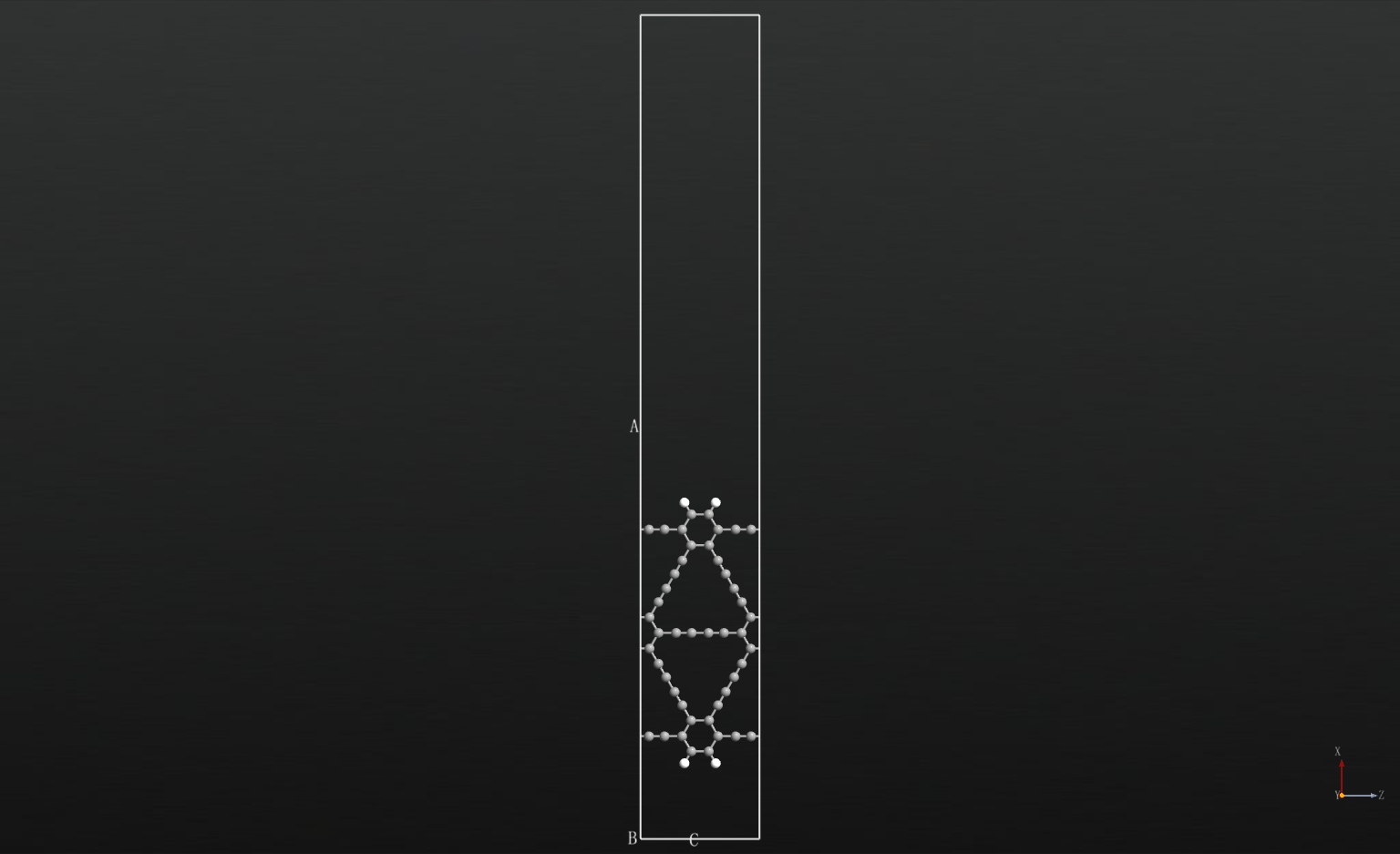

Supplement: Supplementary file 1 — Supplementary Information. [file 41598_2024_56380_MOESM1_ESM.zip › ╓o│┼╨┼╧ó═╝╞1⁄4/Gif S1.gif]

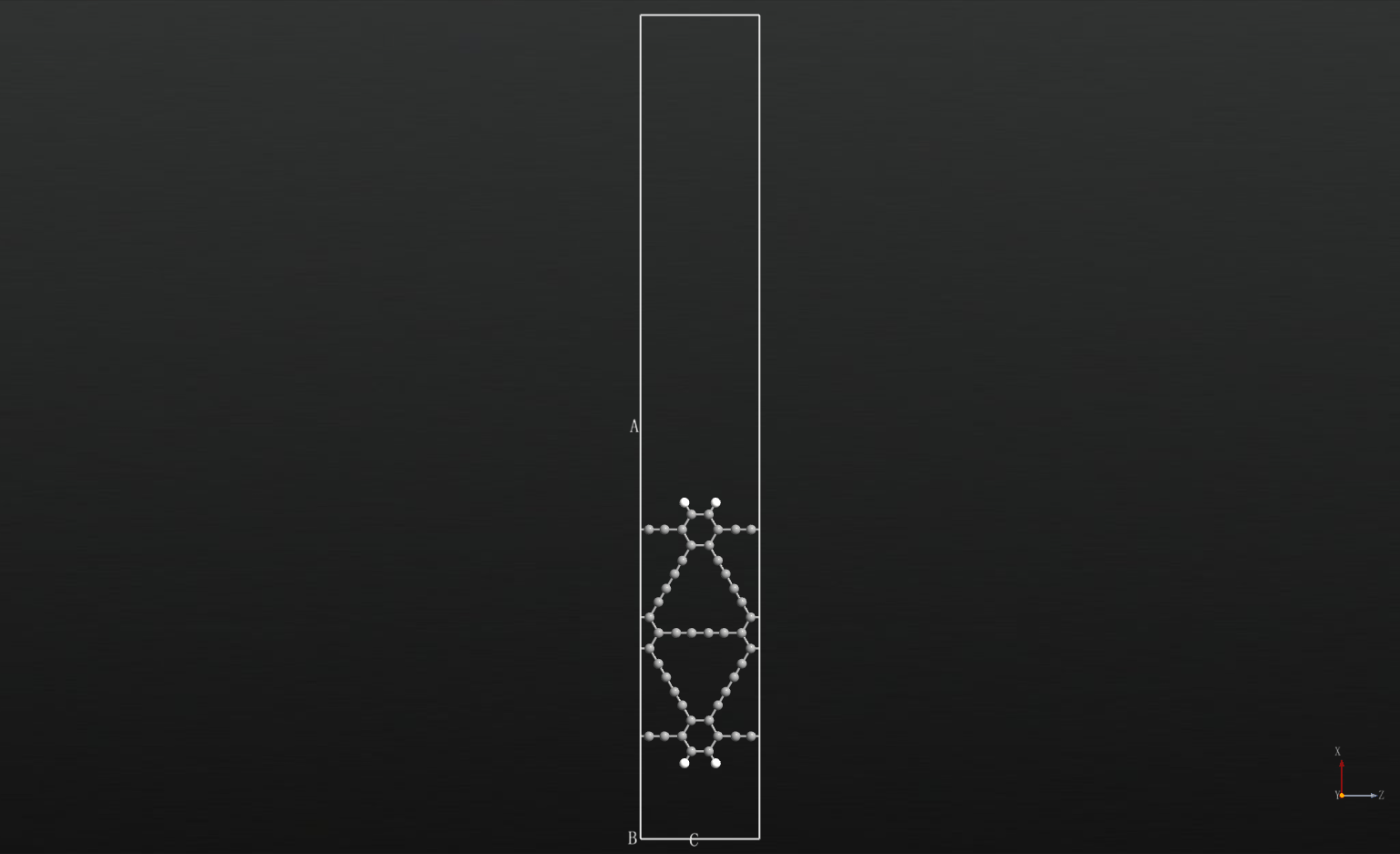

Supplement: Supplementary file 1 — Supplementary Information. [file 41598_2024_56380_MOESM1_ESM.zip › ╓o│┼╨┼╧ó═╝╞1⁄4/Gif S2.gif]

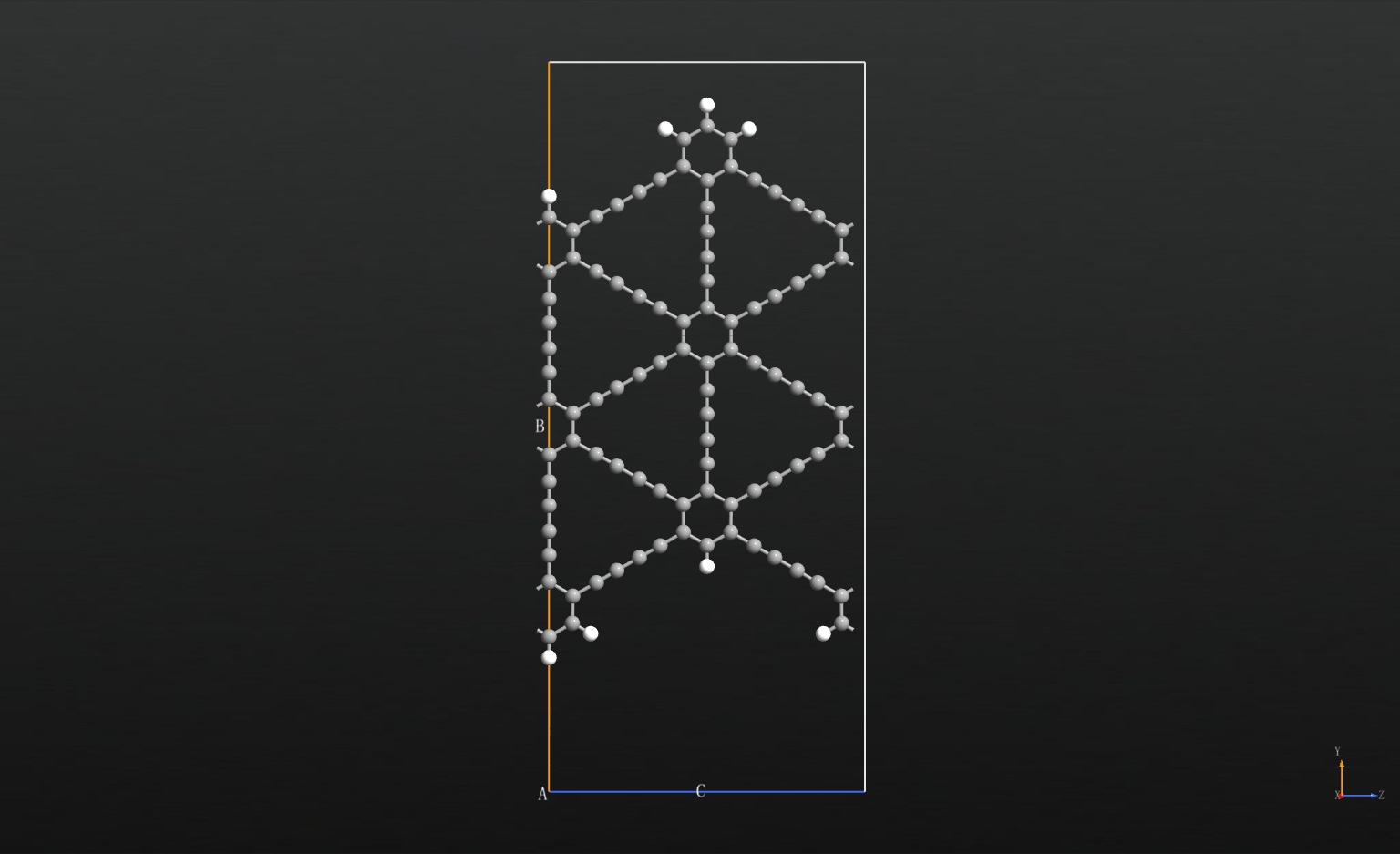

Supplement: Supplementary file 1 — Supplementary Information. [file 41598_2024_56380_MOESM1_ESM.zip › ╓o│┼╨┼╧ó═╝╞1⁄4/Gif S3.gif]

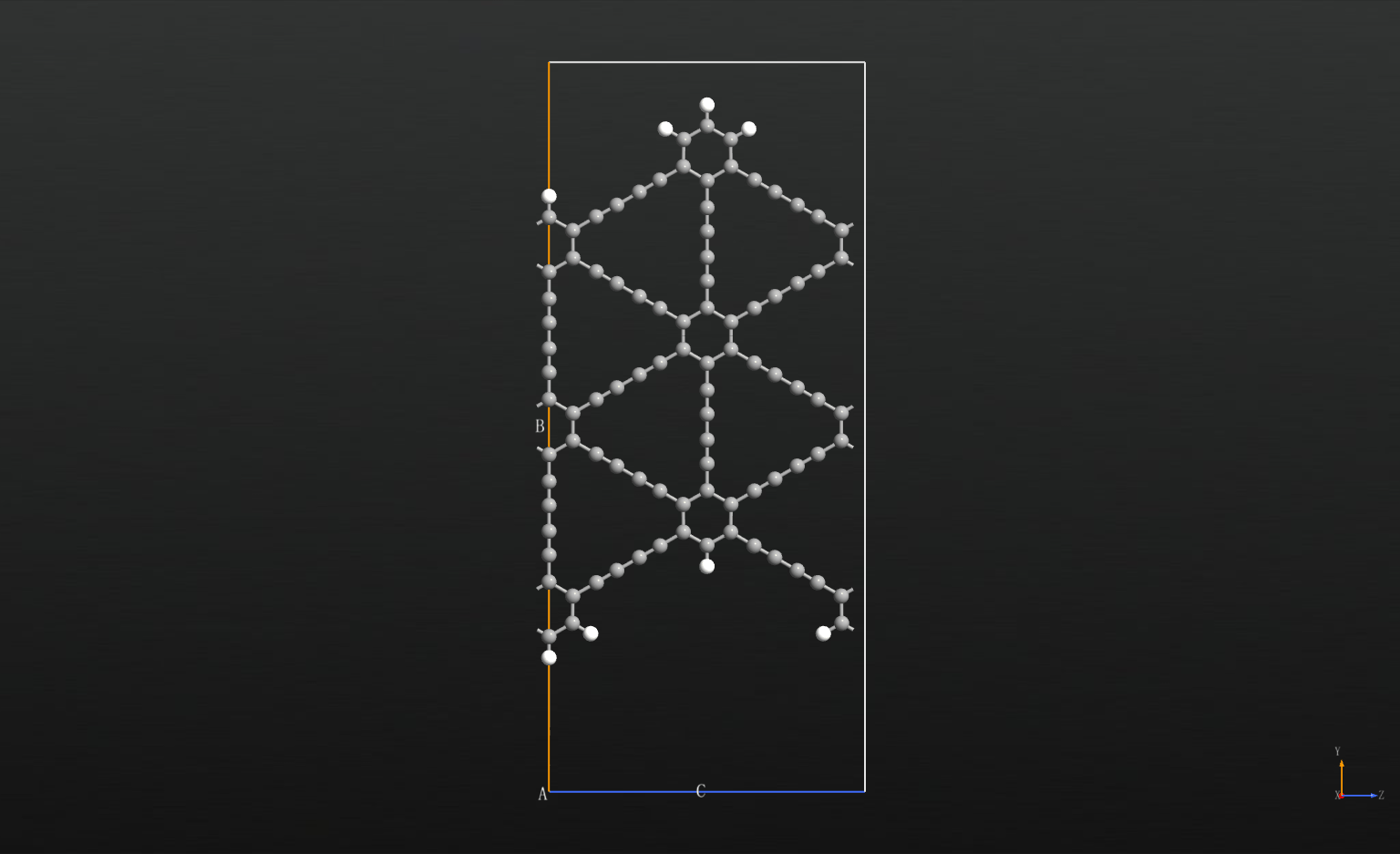

Supplement: Supplementary file 1 — Supplementary Information. [file 41598_2024_56380_MOESM1_ESM.zip › ╓o│┼╨┼╧ó═╝╞1⁄4/Gif S4.gif]
